# Impact of health service interventions on acute hospital use in community-dwelling persons with dementia: A systematic literature review and meta-analysis

**DOI:** 10.1371/journal.pone.0218426

**Published:** 2019-06-21

**Authors:** Claire Godard-Sebillotte, Mélanie Le Berre, Tibor Schuster, Miguel Trottier, Isabelle Vedel

**Affiliations:** 1 Department of Family Medicine, McGill University, Montreal, Quebec, Canada; 2 Lady Davis Institute of Medical Research, Jewish General Hospital, Montreal, Quebec, Canada; 3 Centre de recherche de l’Institut universitaire de gériatrie de Montréal, Université de Montréal, Montreal, Quebec, Canada; Robert Gordon University, UNITED KINGDOM

## Abstract

**Background:**

Persons with dementia have twice the acute hospital use as older persons without dementia. In addition to straining overburdened healthcare systems, acute hospital use impacts patient and caregiver quality of life and is associated with increased risk of adverse outcomes including death. Reducing avoidable acute hospital use in persons with dementia is thus a global healthcare priority. However, evidence regarding the impact of health service interventions as defined by the Effective Practice and Organization of Care Cochrane Group on acute hospital use is scant and inconclusive. The aim of this systematic review and meta-analysis was to synthesize available evidence on the impact of health service interventions on acute hospital use in community-dwelling persons with dementia compared to usual care.

**Methods:**

Data Sources: MEDLINE, EMBASE, CINAHL and Cochrane CENTRAL (from 01/1995 to 08/2017). Study eligibility criteria: Randomised controlled trials measuring the impact of health service interventions on acute hospital use (proportion and mean number of emergency department visits and hospitalisations, mean number of hospital days, measured at 12 months, and at longest follow-up) in community-dwelling persons with dementia, compared to usual care. Study selection, appraisal and synthesis methods: Reviewers independently identified studies, extracted data, and assessed the risk of bias, with the Cochrane risk of bias tool. Authors of relevant trials were queried about unpublished data. Random effects model was used for meta-analyses. Effect heterogeneity was assessed through prediction intervals, and explored using sub-group analyses.

**Findings:**

Seventeen trials provided data on 4,549 persons. Unpublished data were obtained for 13 trials, representing 65% of synthesized data. Most interventions included a case management or a self-management component. None of the outcome comparisons provided conclusive evidence supporting the hypothesis that these interventions would lead to a decrease in acute hospital use. Furthermore, prediction intervals indicated possible and important increased service use associated with these interventions, such as emergency department visits, hospital admissions, and hospital days. Subgroup analyses did not favour any type of intervention. A limitation of this study is the inclusion of any type of health service intervention, which may have increased the observed heterogeneity.

**Conclusion:**

Despite a comprehensive systematic review and meta-analysis, including predominantly unpublished data, no health service intervention beyond usual care was found to reduce acute hospital use in community-dwelling persons with dementia. An important increase in service use may be associated with these interventions. Further research is urgently needed to identify effective interventions for this vulnerable population to limit rising acute hospital use, associated costs and adverse outcomes. Systematic review registration PROSPERO CRD42016046444.

## Introduction

The World Health Organization (WHO) recognizes dementia as the global public health crisis of the 21st century [1,2]. Dementia affects 47.5 million people worldwide and this number is expected to double every 20 years [1]. The dementia population imposes a dramatic strain on healthcare systems worldwide, especially acute hospital use (Emergency Department (ED) visits and hospital admissions). It is estimated that persons with dementia have twice the acute hospital use as older persons without dementia [3–6]. Each year, approximately 40% of community-dwelling persons with dementia will visit the ED and approximately 30% will be hospitalised at least once [4–8]. Hospital care is three times more costly for this population compared to older persons without dementia [9,10]. In addition to straining already overburdened healthcare systems, acute hospital use impacts persons with dementia and their caregiver quality of life and is associated with increased risk of delirium, falls, cognitive and functional decline, 30-day readmission, long-term care admission and death [3–5].

Reducing avoidable hospitalisation and improving health services for persons with dementia are healthcare priorities, as seen in the 2017–2025 WHO action plan [11]. Various health service interventions, including memory clinics or case management, have been designed and implemented over the last two decades to improve practices and organization of care for community-dwelling persons with dementia [12–14]. However, the evidence of impact on acute hospital use is scarce and inconclusive. Previous meta-analyses have focused on case management only, and were unable to show any impact on acute hospital use [14–19]. To date, there is no comprehensive evidence synthesis or meta-analysis on the impact of health service interventions on acute hospital use in community-dwelling persons with dementia [20].

We conducted the first meta-analysis of randomised controlled trials (RCTs) measuring the impact of any type of health service intervention, as defined by the Effective Practice and Organization of Care Cochrane Group, on acute hospital use (ED visits / hospital admissions / hospital days) in community-dwelling persons with dementia compared to usual care.

## Methods

This systematic review and meta-analysis were conducted and reported in accordance with the Cochrane Handbook for Systematic Reviews of Interventions and the Preferred Reporting Items for Systematic Reviews and Meta-Analysis (PRISMA) [21,22]. We followed an a priori registered protocol (PROSPERO ID: CRD42016046444) [23].

### Eligibility criteria

Published articles on RCTs measuring the impact of health service interventions on acute hospital use in community-dwelling persons with dementia were included. **Population:** Community-dwelling persons with dementia or their caregivers or both. **Intervention:** Any health service intervention as defined by the Effective Practice and Organization of Care Cochrane Group (EPOC) Taxonomy 2015: “delivery arrangements”, “financial arrangements”, “governance arrangements”, or “implementation strategies” (Detailed eligibility criteria of interventions in Appendix A in S1 File) [24]. The definition of self-management applied here was the EPOC definition: “Shifting or promoting the responsibility for healthcare or disease management to the patient and/or their family.” (Detailed definition of self-management interventions in Appendix A in S1 File). **Comparison:** Usual care. **Outcomes:** Proportion or mean number of ED visits, proportion or mean number of hospital admissions, mean number of hospital days, in persons with dementia. Eligibility was restricted to interventions in high-income countries [25].

### Information sources and search

We searched for publications in English or French in four databases: MEDLINE (“In-Process & Other Non-Indexed Citations”), EMBASE, CINAHL, and Cochrane Central Register of Controlled Trials (CENTRAL) from January 1995 (first publications on health service interventions for persons living with chronic diseases ^(5)^) to August 2017. The search strategy was developed by a librarian specialized in health service interventions and meta-analysis (MG), a geriatrician (CGS), and an expert in health service interventions for persons with dementia (IV). The key concepts included in the database search were: dementia, health service intervention, community/primary care and RCT (Medline full electronic search strategy in Appendix B in S1 File). Duplicate publications were removed. The search was expanded using backward citation tracking in the reference list of included articles and recent systematic reviews on the topic [14–20] and forward citation tracking of all included studies using Scopus. Authors of relevant trials measuring impact on healthcare costs were inquired about data on acute hospital uses.

### Study selection

Reviewers (CGS, MT, ML) independently assessed all records for eligibility. Disagreements were resolved by consensus or a third reviewer (IV).

### Data collection, transformation and imputation

A systematic approach to data collection, transformation and imputation was followed, as recommended in the Data extraction for complex meta-analysis (DECiMAL) guide (Detailed origin, transformation or imputation of reported data in Appendix C and Tables A-E in S1 File) [26]. Two authors (CGS and ML) independently collected data on structured forms. Companion articles were used if needed, to access data on intervention details. To avoid bias due to selective inclusion of trials effect estimates, corresponding authors of included studies were contacted by email about unpublished data on outcomes at any other time point [27]. In cases of non-response, reminder emails and social media messages (ResearchGate, LinkedIn) were sent to corresponding and last authors. Transformation of data consisted of simple algebraic transformation. Data imputation consisted of weighted mean imputation of missing variance estimates. When the sample size in each group was not clearly stated, the randomised number of individuals in the text or flow chart determined the intention-to-treat population. Clustered randomised trials were identified and data adjusted on the clustering effect was collected. If unavailable, unadjusted data was collected.

### Quality appraisal

Risk of bias was rated at the study level by two independent reviewers (CGS and ML), using the Cochrane Collaboration’s tool [28]. Companion articles, especially published protocols, were used to appraise quality. Blinding of participants and personnel was not assessed due to the nature of the interventions. For the six remaining individual domains, studies were classified into low, unclear or high risk of bias according to specific criteria of the tool [28]. As recommended by the Cochrane Handbook for Systematic Reviews of Interventions, studies of low quality were not excluded, but sensitivity analyses were conducted [21].

### Summary measures

We pooled estimates for the following five outcomes at two endpoints. We pooled the estimated proportions of persons having at least one ED visit and/or at least one hospital admission, the mean number of ED visits, the mean number of hospital admissions, and the mean number of hospital days on the total sample of participants irrespective of whether participants used the corresponding service. Following the recommendations of the Cochrane Handbook for Systematic Reviews of Interventions on repeated observations on participants, we compiled data available for each outcome at 12 months and at longest follow-up for each study [21].

### Synthesis

The statistical software R, and the meta and ggplot2 packages, were used to perform analyses [29]. The unit of analysis was the unique RCT. Random-effects models were employed to allow for varying effect sizes across studies due to heterogeneity of interventions and/or study populations. Risk differences, risk ratio and mean differences were calculated to determine the average relative and absolute effect of the interventions on the dichotomous and continuous outcomes. Not every cluster RCT study had published data adjusting for a potential clustering effect. Mixing unadjusted data from cluster RCTs with data from individual RCTs can lead to artificially narrow confidence intervals [30]. The Cochrane Collaboration recommends performing an “effective sample size” calculation to pool unadjusted data from cluster RCTs and individual RCTs together. As data necessary to perform this ‘effective sample size’ calculation was not available for every cluster RCT, we performed sensitivity analyses excluding unadjusted data from cluster RCTs [30]. Heterogeneity across studies was assessed by calculating the I^2^ statistic as well as predictions intervals. Following Cochrane recommendations, our interpretation of the I^2^ statistic was that over 40% may represent moderate to considerable heterogeneity [21]. Prediction intervals are another measure of heterogeneity and are easier to interpret and relate to the clinical implication of the observed heterogeneity [31]. Prediction intervals estimate a pre-specified distribution range (here: 95%) of treatment effects that can be expected in future settings. Meta-analysis results were labelled inconclusive if the range of treatment effects consistent with the prediction interval included both positive and negative clinically relevant effects.

Post hoc subgroup analyses were performed to explore heterogeneity for outcomes pooling a minimum of four studies and showing moderate to considerable heterogeneity (I^2^ > 40%). Criteria used to perform these subgroup analyses were the types of interventions according to the EPOC taxonomy (either the main component or one of several), country (United States vs. other), and follow-up time. The number of studies included did not allow meaningful application of meta-regression methods.

We conducted several sensitivity analyses to determine the robustness of meta-analysis results. We investigated changes in estimated pooled effects when removing: RCTs with at least one item at high risk of bias, outlying RCTs based on a graphical assessment of the corresponding forest plot, and cluster RCTs that did not properly take clustering into account. We did not generate funnel plots to identify reporting bias, because interpretation would have been questionable since most of the data in the analysis was unpublished. We conducted additional sensitivity analyses investigating changes in estimated pooled effects due to removing unpublished data provided by authors from the analyses [32].

## Results

### Studies characteristics

**Fig 1:**
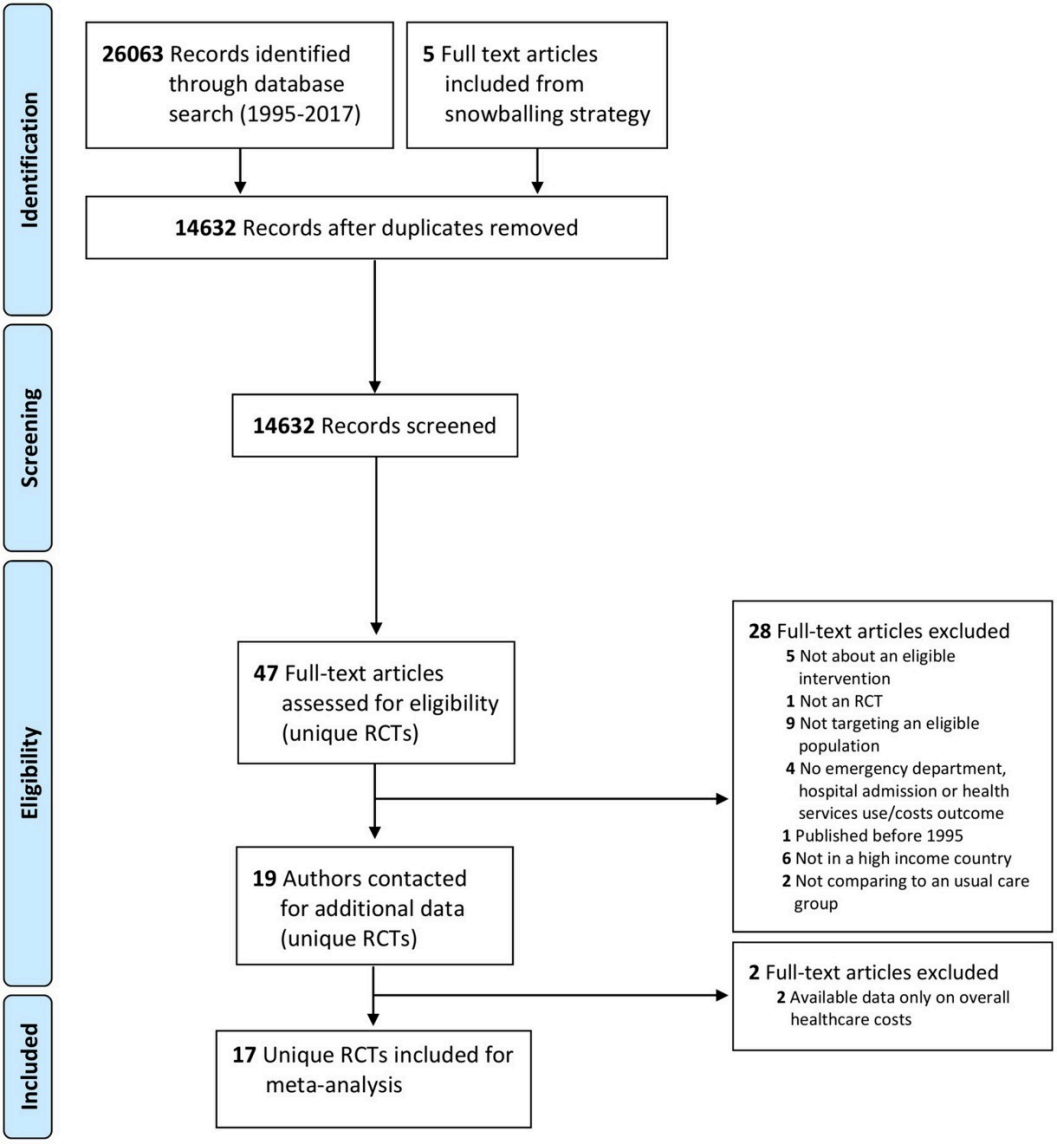
Preferred reporting items for systematic reviews and meta-analysis (PRISMA) flow diagram of study selection. Fig 1 legend: RCT, Randomised controlled trial.

The systematic literature search resulted in 19 eligible, unique RCTs (Fig 1). Two of these were not included as the available data was only on overall healthcare costs (confirmed by study authors) [33,34]. Seventeen unique RCTs were included in the meta-analyses including four cluster RCTs (Table 1). Eight of 17 RCTs were included because unpublished data was provided by authors [13,35–41]. The published data was either only cost data or combined outcomes on use (e.g. long term care admission and hospitalisation) [13,35–41]. We obtained unpublished data for thirteen trials on five outcomes measured at two endpoints, representing 65% of overall synthesized data (Detailed origin, transformation or imputation of reported data in Appendix C and Tables A-E in S1 File).

**Table 1:** Studies characteristics.

Authors Publication Date	Country	Intervention type EPOC taxonomy (main component in bold)	Intervention duration	Type of neurocognitive disorder and severity (MMSE if available)	Sample size^a^	Age, Mean (SD)^b^	Female No (%)^b^
**Callahan** **2006** **[42]**	United States	**Teams**/case management/self-management/use of information and communication technology	maximum of 12 months	AD; “moderate severity” (mean MMSE: 18)	I: 84; C: 69	I: 77 (6); C: 78 (6)	I: 39 (46); C: 27 (39)
**Bass 2003** **[43,44]**	United States	**Self-management**/case management	12 months	Dementia diagnosis or a symptom code indicating memory loss; severity not reported	I:72; C:48	Not reported	Not reported
**Challis 2004** **[45]**	United Kingdom	**Comprehensive geriatric assessment**	6 months	MMSE lower than 24 (I: 67%; C: 54%); severity not reported	I: 129; C: 127	I: 82 (7); C: 82 (8)	I: 96 (74); C: 92 (72)
**Chien 2008** **[36]**	Hong Kong	**Self-management**/case management	6 months	AD (DSM IV criteria); 80% of the sample at “early (ambulatory) stage"	I: 44; C: 44	Total sample: 68 (7)	Total sample: 38 (43)
**Chien 2011** **[37]**	Hong Kong	**Self-management**/case management	24 months	AD (DSM IV criteria); “mild or moderate” severity (mean MMSE: I:18, C:17)	I: 46; C: 46	I: 68 (7); C: 67 (7)	I: 19 (41); C: 21 (46)
**Duru 2009** **[46,47]**	United States	**Case management**/self-management/use of information and communication technology/educational material and educational meetings (healthcare professionals’ education)	18 months	AD, vascular dementia and other types of dementia; (Blessed-Roth Dementia Scale mean scores: I: 5, C: 6)	I: 238; C: 170	I: 79 (6); C: 80 (7)	I: 94 (55); C: 71 (56)
**Eloniemi-Sulkava 2009** **[40]**	Finland	**Teams**/comprehensive geriatric assessment/case management/self-management	24 months	AD, vascular dementia and other types of dementia; “mild”, “moderate” and “severe” dementia (mean MMSE: 14)	I: 63; C: 62	I: 78 (7); C: 77 (6)	I: (43) ^c^; C: (32)^c^
Joling 2013 [38,48,49]	Netherlands	**Self-management**	12 months	Dementia diagnosis; (mean MMSE: I: 21, C: 22)	I: 96; C: 96	I: 73 (9); C: 77 (8)	I: 30 (31); C: 32 (33)
Laakkonen 2016 [41,50]	Finland	**Self-management**	8 weeks	Dementia diagnosis; “possible”, “mild”, “moderate” and “severe” dementia (mean MMSE: I: 20, C: 22)	I: 67; C: 69	I: 77 (6); C: 77 (6)	I: 25 (37); C: 26 (38)
Meeuwsen 2013 [51–53]	Netherlands	**Site of service delivery** (memory clinic vs general practitioner)/teams	12 months	AD, vascular dementia, other types of dementia; “very mild” and “mild” dementia (mean MMSE: 23)	I: 87; C: 88	I: 78 (6); C: 78 (5)	I: 54 (62); C: 52 (59)
Menn 2012 [39,54,55]	Germany	**Self-management**/educational material and educational meetings (healthcare professionals’ education)/shared care	24 months	Dementia diagnosis;”mild” and “moderate” dementia (mean MMSE: I-groupB: 19, I-groupC: 19, C: 18)	I-groupB: 109 I-groupC: 110 C: 171	I-groupB: 79 (6); I-groupC: 81 (6); C: 81 (7)	I-groupB: (68) ^c^; I-groupC: (71) ^c^; C: (67) ^c^
Nichols 2017 [35,56]	United States	**Self-management**	6 months	AD, Dementia diagnosis or MMSE lower than 24; severity not reported	I: 98; C: 99	I: 80 (8); C: 78 (9)	I: 59 (60); C: 59 (60)
Rubenstein, 2007 [57]	United States	**Case management**/teams	36 months	Cognitive impairment (10-item Geriatric Postal Screening Survey); severity not reported	I: 380; C: 412	I: 75 (6); C: 74 (6)	I: 14 (4); C: 11 (3)
Samus 2014 [58,59]	United States	**Teams**/case management/self-management/use of information and communication technology	18 months	Dementia or “Cognitive Disorder Not Otherwise Specified” (DSM IV criteria); “mild”, “moderate” and “severe” dementia (mean MMSE: 19)	I: 110; C: 193	I: 84 (6); C: 84 (6)	I: 73 (66); C:120 (62)
Søgaard 2014 [60–62]	Denmark	**Self-management**	36 months	AD, mixed dementia, or Lewy body dementia; “mild” dementia (mean MMSE: 24)	I: 163; C: 167	I: 76 (8); C: 75 (7)	I: 87 (53); C: 92 (55)
Thyrian 2017 [13,63,64]	Germany	**Case management**/ use of information and communication technology /teams	12 months	Positive screening for dementia (DemTect procedure); “no hint for”, “mild”, “moderate” and “severe” dementia (mean MMSE: 23)	I: 408; C: 226	I: 81 (6); C: 80 (5)	I: 178 (61); C: 70 (60)
Wray 2010 [65]	United States	**Self-management**	10 weeks	Diagnosis of dementia; "moderate-to-severe" dementia	I: 83; C: 75	I: 78 (7); C: 79 (8)	Not reported

Abbreviations: AD, Alzheimer’s Disease; DSM IV, Diagnostic and Statistical Manual of Mental Disorders IV; EPOC, Effective Practice and Organization of Care Cochrane Group; MMSE, Mini-Mental State Examination; I, Intervention Group; C, Control Group

^a^ The numbers are the randomised numbers of participants in each group (as reported or calculated).

^b^ Denominators are the number of participants with available baseline characteristics. They differ from the randomised numbers of participants for two trials: Thyrian 2017 and Duru 2009. In Thyrian 2017, the numbers of participants with available baseline characteristics are 291 in intervention and 116 in control groups. In Duru 2009, the numbers of participants with available baseline characteristics are 170 in intervention and 126 in control groups.

^c^ Only percentages of female participants were reported.

Seventeen unique trials provided data on 4,549 community-dwelling persons living with dementia (study populations ranging from 88 to 792 persons, median randomisation arm size: 96) (Table 1) ^(13.36–65)^. These persons had a mean age of 77 years (standard deviation: 4) and 49% were females (proportion ranged from 4% to 74%). Twelve of the seventeen trials reported dementia severity at baseline, ranging from mild to severe: 6/12 mild, 5/12 moderate, 1/12 moderate to severe. None of the trials reported selection of participants based on risk of acute hospital use.

Health service interventions consisted of one or a combination of the following EPOC taxonomy components: case management (9/17), self-management (13/17), comprehensive geriatric assessment (2/17), educational materials/educational meetings (healthcare professional education) (2/17), use of information and communication technology (4/17), teams (6/17), shared care (1/17), site of service delivery (memory clinic) (1/17). None of the interventions involved financial or governance arrangements. Interventions were implemented and evaluated in studies conducted in the USA (7), in Europe (8) and in Hong Kong (2). Duration of interventions ranged from two to 36 months, median duration 12 months.

The sources of the data for each outcome measure included in the meta-analysis are presented in Table 2.

**Table 2:** Intervention types and outcomes included in the meta-analysis for each study.

Authors Publication Date	Intervention type EPOC taxonomy (main component in bold)	Outcomes				
		Proportion of patients with at least one ED visit	Mean number of ED visits	Proportion of patients with at least one hospital admission	Mean number of hospital admissions	Mean number of hospital days
Callahan 2006 [42]	**Teams**/case management/self-management/use of information and communication technology			✓		✓
Bass 2003 [43,44]	**Self-management**/case management		✓		✓	
Challis 2004 [45]	**Comprehensive geriatric assessment**	✓	✓	✓		✓
Chien 2008 [36]	**Self-management**/case management	✓		✓	✓	✓
Chien 2011 [37]	**Self-management**/case management	✓		✓	✓	✓
Duru 2009 [46,47]	**Case management**/self-management/use of information and communication technology/ educational material and educational meetings (healthcare professionals’ education)	✓	✓	✓	✓	
Eloniemi-Sulkava 2009 [40]	**Teams**/comprehensive geriatric assessment/case management/self-management			✓		✓
Joling 2013 [38,48,49]	**Self-management**			✓		✓
Laakkonen 2016 [41,50]	**Self-management**			✓		✓
Menn 2012 [39,54,55]	**Self-management**/educational material and educational meetings (healthcare professionals’ education)/shared care			✓	✓	✓
Nichols 2017 [35,56]	**Self-management**	✓	✓	✓	✓	✓
Rubenstein, 2007 [57]	**Case management**/teams			✓		✓
Samus 2014 [58,59]	**Teams**/case management/self-management/use of information and communication technology	✓	✓	✓	✓	✓
Søgaard 2014 [60–62]	**Self-management**	✓	✓	✓	✓	✓
Thyrian 2017 [13,63,64]	**Case management**/ use of information and communication technology /teams	✓	✓	✓	✓	✓
Wray 2010 [65]	**Self-management**				✓	✓

Abbreviations: EPOC, Effective Practice and Organization of Care Cochrane Group.

### Impact on acute hospital use

**Fig 2:**
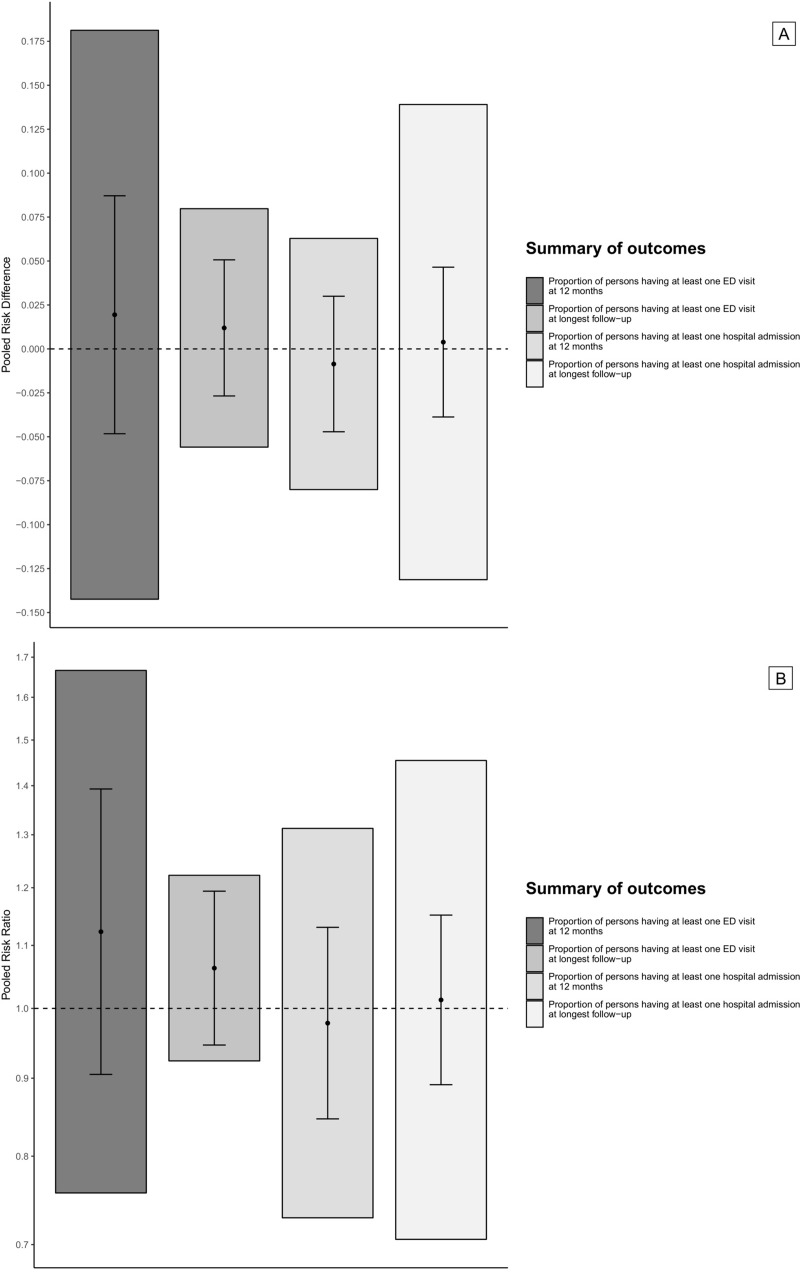
**Pooled risk differences (2A) and pooled risk ratios (2B) for dichotomous outcomes (solid dots), 95% confidence intervals (black coloured error bars), and 95% prediction intervals (grey shaded bar plots).** Fig 2 legend: ED, Emergency Department.

**Fig 3:**
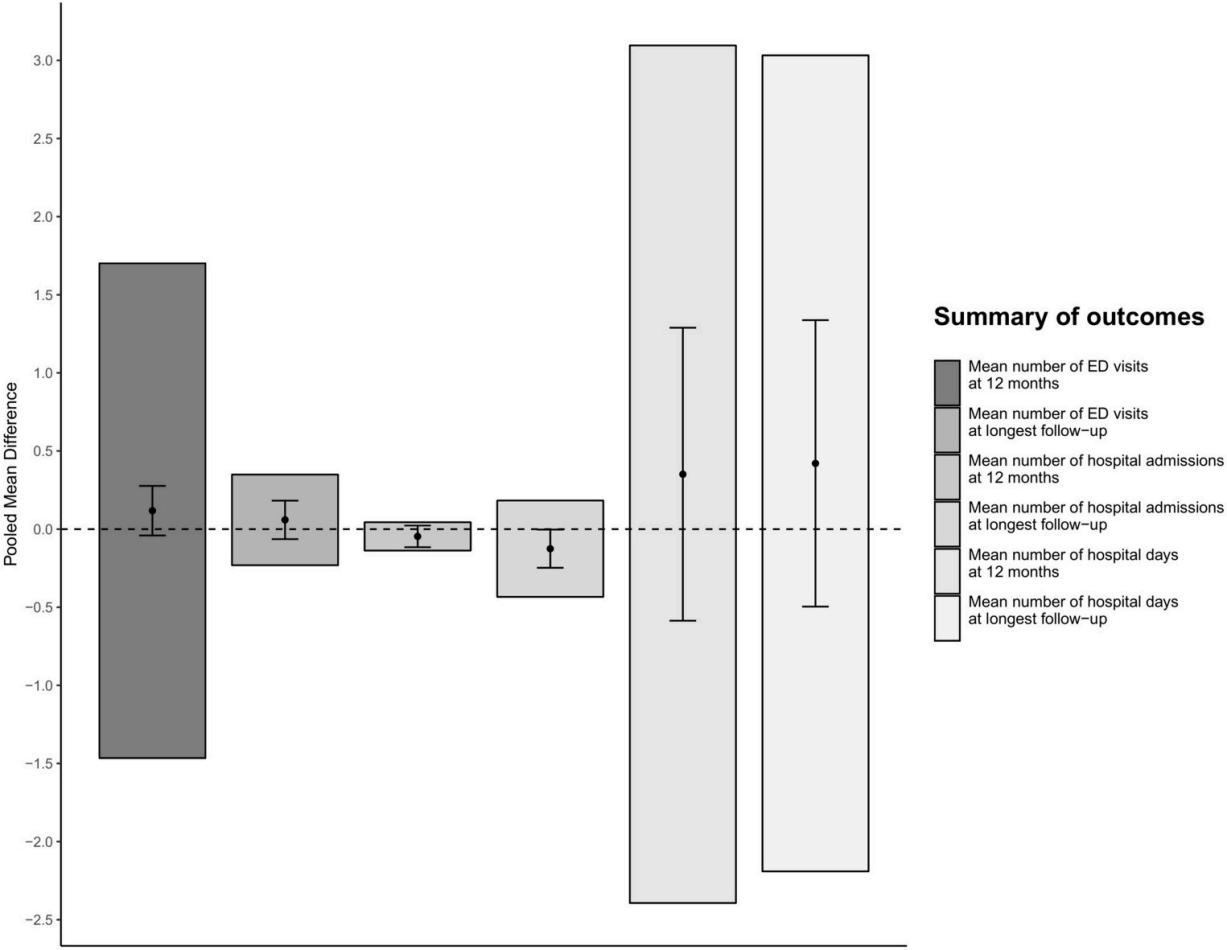
Pooled mean differences for continuous outcomes (solid dots), 95% confidence intervals (black coloured error bars), and 95% prediction intervals (grey shaded bar plots). Fig 3 legend: ED, Emergency Department.

None of the considered outcome comparisons provided conclusive evidence supporting the hypothesis that health service interventions lead to a decrease in service use as measured by ED visits, hospital admission or hospital days (Figs 2 and 3 and Figs A-E in S1 File). Furthermore, in every meta-analysis, the estimated 95% prediction intervals indicated that an important increase in service use may be associated with the interventions.

Post hoc subgroup analyses did not suggest any systematic dependencies of effects in relation to type of intervention, country or follow-up time.

### Quality and robustness of the evidence

Four of the 17 trials were judged as having at least one area of high risk of bias (Fig F in S1 File). Eleven trials reported adequate sequence generation, 11 trials properly concealed allocation, 14 trials implemented blinding of outcome assessors, and 15 trials adequately addressed incomplete outcome data. Only eight trials published a study protocol and reported (or provided upon request) pre-specified outcomes.

Sensitivity analyses to determine the robustness of meta-analysis results did not lead to any change in estimated pooled effects that would alter the conclusions. Sensitivity analyses of the impact of unpublished data provided by authors on meta-analysis results did not suggest reporting bias.

## Discussion

The results of this systematic review and meta-analysis did not establish superiority of health service interventions over usual care to reduce acute hospital use in community-dwelling persons with dementia. There was no detectable signal favouring any type of health service intervention. Overall evidence had low risk of bias. Sensitivity analyses confirmed the robustness of the results. Furthermore, for every outcome, the estimated 95% prediction intervals indicated that an important increase in service use may be associated with these interventions.

These results are of particular interest to policy makers, persons living with dementia, their caregivers, and healthcare professionals. There is no cure or disease-modifying treatment for dementia and no promising advances in the near future [66]. Improving healthcare delivery remains essential to limit rising acute hospital use and associated costs, improve quality of life of patients and their caregivers, and prevent adverse outcomes for persons living with dementia. The absence of evidence for an impact on acute hospital use of any type of health service intervention is thus highly concerning. The possibility that these interventions may increase acute hospital use is not to be disregarded.

Non-intended effects of health service interventions, such as increase in service use (ED visits, hospital admissions, and hospital days) have previously been witnessed in several contexts, such as self-management interventions. These non-intended effects were associated with either beneficial or adverse outcomes. Some health service interventions, like self-management interventions for caregivers, have led to non-intended beneficial outcomes: increased service use due to increased caregiver awareness of symptoms, diagnosis procedures and treatment options [38,60]. Some health service interventions, such as self-management in patients with chronic obstructive pulmonary disease (COPD), have led to non-intended adverse outcomes. A decreased service use due to patient overconfidence in self-management led to higher mortality [67].

It is essential to better characterize acute hospital use and inappropriate use, so that beneficial and adverse non-intended outcomes can be sorted out. In the trials included in this systematic review and meta-analysis, only total acute hospital use was measured. However, community-dwelling persons with dementia would have a much greater chance of potentially avoidable hospitalizations (74%) or ED visits (51%), than persons without dementia [5]. Definitions and measures of potentially avoidable acute hospital use such as Ambulatory-Care Sensitive Conditions (ACSC) hospital admissions have recently been questioned for inaccuracy in community-dwelling persons living with dementia [68]. Developing accurate measures of potentially avoidable service use in community-dwelling persons living with dementia is essential [68].

Two main reasons could explain the inconclusive results of this evidence synthesis. First, the included RCTs might not have detected an effect because of lack of statistical power. Lack of power could be a consequence of inappropriate specification of the target population. These RCTs did not exclusively consider high-risk populations for acute hospital use. Targeting high-risk populations may be necessary to show measurable reductions in acute hospital use due to health service interventions. To our knowledge, no screening tool is available to identify community-dwelling persons with dementia with high-risk of acute hospital use, so its development is essential [69].

Second, there might be a gap between the focus of the interventions and the actual causes of acute hospital use [20,70]. The interventions may not have effectively addressed the causes of acute hospital use of community-dwelling persons with dementia [20,70,71]. Only a few types of interventions were tested in the 17 trials, mainly case management and self-management. Case management would have been effective if the reasons for acute hospital use were care fragmentation. However, care fragmentation was not identified as a major determinant of crises leading to acute hospital use [70,72]. Since behavioural and psychological symptoms of dementia are not leading causes of hospital admissions, increasing self-management skills for patients and caregivers would not have been effective [3,71].

The literature suggests that physical conditions are a leading cause of acute hospital use in persons with dementia. Most admissions are due to accidents and injuries arising from falls, urinary tract and respiratory infections, or complications of chronic diseases [3,70–72]. Improving access to primary health care and training home-care staff on early detection and appropriate management of the common causes of acute hospital use could reduce avoidable acute hospital use [68,73].

Caregiver availability and caregiver health are other important determinants of acute hospital use. Indeed, caregiver stress, burden, mental health and sudden absence (hospital admission or death) are identified as major drivers of crises [70–72,74]. Offering timely support to caregivers through respite care or temporary home care could reduce avoidable acute hospital use [20].

During the final year of life, nearly 80% of community-dwelling persons with dementia are hospitalized [5]. Some of these hospitalizations may not be the choice of patients and caregivers, who may have preferred to obtain end-of-life care at home [75]. Palliative care in older persons with advanced illness has been shown to double the chances of dying at home [76]. Interventions emphasizing a palliative care approach with discussion of advanced directives and preferences for end-of-life care might reduce undesired acute hospital use.

### Strengths and limits

Our study has strengths as well as potential limitations. This is the first systematic review and meta-analysis on the impact of any type of health service interventions on acute hospital use in community-dwelling persons with dementia, and the first to include predominantly unpublished data provided by the authors of included randomised controlled trials. Two main challenges were encountered when retrieving data for this synthesis: i) some evidence was published as cost-effectiveness analysis; ii) outcomes were measured in different ways. We gathered data pro-actively by contacting authors of identified trials, and used a systematic approach for data transformation and imputation. These strategies dramatically increased the range of synthesised evidence and were likely to have decreased potential publication bias impact on our effect estimates [77,78].

Acknowledging the complexity of acute hospital use prevention in this vulnerable population, we included any type of health service intervention as defined and classified in the Cochrane EPOC taxonomy [24]. This is a common approach in Cochrane reviews [79], but might have increased the observed heterogeneity. We computed prediction intervals and performed sub-group analyses to explain possible sources of effect heterogeneity. We used prediction intervals to conservatively interpret the range of expected treatment effects in future studies rather than an average effect composed by a set of different underlying effects. Our sub-group analyses were based on limited descriptions provided in the articles, which limited our understanding of heterogeneity [80]. For example, usual care and primary care access might vary widely between countries, regions, or subpopulations and were rarely described in the studies. Likewise, intervention descriptions were sometimes too limited to classify interventions according to the EPOC taxonomy. We thus looked for protocols and companion articles and performed independent data extraction to reduce subjectivity.

## Conclusion, policy implications and future research

With the data available, it was not possible to establish superiority of any health service intervention beyond usual care to reduce acute hospital use in community-dwelling persons with dementia. In fact, our evidence synthesis findings do not rule out the possibility that the studied health service interventions may be associated with an important increase in service use.

We have no recommendations for health service interventions to be implemented. However, we can propose a research agenda focused on: 1) development of accurate measures of potentially avoidable acute hospital use by community-dwelling persons with dementia; 2) identifying the causes and determinants of potentially avoidable acute hospital use; 3) development of a validated screening tool to target high-risk population; 4) co-design of health service interventions with patients and caregivers that address the causes of avoidable acute hospital use; and 5) rigorous testing of the impact of these co-designed interventions in high-risk community-dwelling persons with dementia.

## Supporting information

S1 File Supporting information. S1 file legend:
Appendix A: Detailed eligibility criteria of intervention.
Appendix B: Medline full electronic search strategy.
Appendix C: Detailed origin, transformation or imputation of reported data.
Table A: Origin, transformation or imputation of data for proportions of persons having at least one Emergency Department visit.
Table B: Origin, transformation or imputation of data for mean number of Emergency Department visit.
Table C: Origin, transformation or imputation of data for proportions of persons having at least one hospital admission.
Table D: Origin, transformation or imputation of data for mean number of hospital admission.
Table E: Origin, transformation or imputation of data for mean number of hospital days.
Fig A. Proportion of persons having at least one Emergency Department visit (Risk Ratio and Risk Difference). A. At 12 months. B. At the longest follow-up. Abbreviations: CI, Confidence Interval; RD, Risk Difference; RR, Risk Ratio.
Fig B. Proportion of persons having at least one hospital admission (Risk Ratio and Risk Difference). A. At 12 months. B. At the longest follow-up. Abbreviations: CI, Confidence Interval; RD, Risk Difference; RR, Risk Ratio.
Fig C. Mean number of Emergency Department visit (Mean Difference). A. At 12 months. B. At the longest follow-up. Abbreviations: CI, Confidence Interval; MD, Mean Difference; SD, Standard Deviation.
Fig D. Mean number of hospital admission (Mean Difference). A. At 12 months. B. At the longest follow-up. Abbreviations: CI, Confidence Interval; MD, Mean Difference; SD, Standard Deviation.
Fig E. Mean number of hospital days (Mean Difference). A. At 12 months. B. At the longest follow-up. Abbreviations: CI, Confidence Interval; MD, Mean Difference; SD, Standard Deviation.
Fig F. Quality Appraisal using the Cochrane Risk of Bias Tool. a Random sequence generation (selection bias). b Allocation concealment (selection bias). c Blinding of participant and personnel (performance bias). d Blinding of outcome assessment. e Incomplete outcome data (attrition bias). f Selective reporting (reporting bias). g Other bias. h Questions marks, uncertain risk of bias; + signs, low risk of bias;—signs, high risk of bias.
References.
(DOCX)

S2 File PRISMA checklist. (DOCX)
