# Supplementary material for: Impact of health service interventions on acute hospital use in community-dwelling persons with dementia: A systematic literature review and meta-analysis
Source: PLoS One. 2019 Jun 21;14(6):e0218426. doi: 10.1371/journal.pone.0218426 (PMC6588225; doi:10.1371/journal.pone.0218426)
Supplement: S1 File — legend: Appendix A: Detailed eligibility criteria of intervention. Appendix B: Medline full electronic search strategy. Appendix C: Detailed origin, transformation or imputation of reported data. Table A: Origin, transformation or imputation of data for proportions of persons having at least one Emergency Department visit. Table B: Origin, transformation or imputation of data for mean number of Emergency Department visit. Table C: Origin, transformation or imputation of data for proportions of persons having at least one hospital admission. Table D: Origin, transformation or imputation of data for mean number of hospital admission. Table E: Origin, transformation or imputation of data for mean number of hospital days. Fig A. Proportion of persons having at least one Emergency Department visit (Risk Ratio and Risk Difference). A. At 12 months. B. At the longest follow-up. Abbreviations: CI, Confidence Interval; RD, Risk Difference; RR, Risk Ratio. Fig B. Proportion of persons having at least one hospital admission (Risk Ratio and Risk Difference). A. At 12 months. B. At the longest follow-up. Abbreviations: CI, Confidence Interval; RD, Risk Difference; RR, Risk Ratio. Fig C. Mean number of Emergency Department visit (Mean Difference). A. At 12 months. B. At the longest follow-up. Abbreviations: CI, Confidence Interval; MD, Mean Difference; SD, Standard Deviation. Fig D. Mean number of hospital admission (Mean Difference). A. At 12 months. B. At the longest follow-up. Abbreviations: CI, Confidence Interval; MD, Mean Difference; SD, Standard Deviation. Fig E. Mean number of hospital days (Mean Difference). A. At 12 months. B. At the longest follow-up. Abbreviations: CI, Confidence Interval; MD, Mean Difference; SD, Standard Deviation. Fig F. Quality Appraisal using the Cochrane Risk of Bias Tool. a Random sequence generation (selection bias). b Allocation concealment (selection bias). c Blinding of participant and personnel (performance bias). d Blinding of out [file pone.0218426.s001.docx]

# S1 File. Supporting information

# Appendix A: Detailed eligibility criteria of intervention

Any health services intervention as defined and classified by the Effective Practice and Organization of Care Cochrane Group (EPOC) Taxonomy 2015: “delivery arrangements”, “financial arrangements”, “governance arrangements”, or “implementation strategies” ^(1).^

Delivery arrangements changes includes, but are not limited to

- changes in where the healthcare is provided and changes to the healthcare environment (e.g. respite care, memory clinic),
- changes in who is providing care and how the workforce is managed (e.g. self-management), coordination of care and management of care processes (e.g.: case management, interdisciplinary team, comprehensive geriatric assessment, care transition, liaison with community organizations (e.g. home care, Alzheimer’s Society), disease management (decision support tools (protocol, algorithm),
- Information and communication technology (e.g.: health information systems, smart home technology, telemedicine).

Financial arrangements changes include, but are not limited to, changes in out of pocket payments, and financial incentives.

Implementation strategies include, but are not limited to, educational meetings, educational materials targeted at healthcare workers, inter-professional education, and local opinion leaders.

As there is currently no consensual definition of what a self-management intervention in dementia is, and to whom should it be delivered: the caregiver or the care recipient, included interventions were those corresponding to the EPOC definition of self-management: “Shifting or promoting the responsibility for healthcare or disease management to the patient and/or their family.” Included interventions of self-management were thus aimed at the caregiver or the patient or both. Reminiscence groups were excluded as they often do not provide group members with new skills in problem solving and goal setting. Therapy (e.g. cognitive behavioural therapy, occupational therapy) directed to the care recipient or the caregiver was also excluded even if these interventions may help coping with caregiving or the disease, since their primary aim is to provide therapy rather than provide skills or social support. We excluded supports groups for the caregiver or the patients, when they were not including any component of a self-management intervention and just providing emotional support.

# Appendix B: Medline full electronic search strategy

Database: Ovid MEDLINE(R) In-Process & Other Non-Indexed Citations, Ovid MEDLINE(R) Daily, Ovid MEDLINE(R) and Ovid OLDMEDLINE(R) <1946 to Present>

Search Strategy:

--------------------------------------------------------------------------------

1 dementia/ or aids dementia complex/ or alzheimer disease/ or aphasia, primary progressive/ or primary progressive nonfluent aphasia/ or creutzfeldt-jakob syndrome/ or dementia, vascular/ or cadasil/ or dementia, multi-infarct/ or diffuse neurofibrillary tangles with calcification/ or frontotemporal lobar degeneration/ or frontotemporal dementia/ or huntington disease/ or kluver-bucy syndrome/ or lewy body disease/ or "pick disease of the brain"/

2 cognition disorders/ or auditory perceptual disorders/ or huntington disease/ or mild cognitive impairment/

3 ((cogn* adj1 disorder?) or (cogn* adj1 impairment?)).mp.

4 (dementia? or alzheimer*).mp.

5 1 or 2 or 3 or 4

6 patient care management/ or comprehensive health care/ or exp patient care planning/ or case management/ or exp patient-centered care/ or exp critical pathways/ or "delivery of health care"/ or exp after-hours care/ or exp "delivery of health care, integrated"/ or exp health services accessibility/ or exp disease management/ or exp patient care team/ or patient-centered care/ or "quality of health care"/ or exp "outcome and process assessment (health care)"/ or "utilization review"/

7 ((interdisciplin* or multidisciplin*) adj2 team?).mp.

8 (enhanc* adj access*).ti,ab.

9 (memory adj clinic?).mp.

10 ((care adj coordinat?) or (care adj transit?) or (care adj manag*) or (case adj manag*) or (care adj navigator?) or (critical adj pathway?) or (clinical adj pathway?) or (continu* adj1 care) or (disease? adj management)).mp.

11 intervention*.ti,ab.

12 exp Program Development/

13 (program adj (development or evaluation)).mp.

14 self care/ or patient education as topic/ or consumer participation/

15 (self-mangement or "self management" or self?management).ti,ab.

16 exp Geriatric Assessment/

17 Follow-Up Studies/

18 exp Models, Organizational/

19 model*.ti,ab.

20 6 or 7 or 8 or 9 or 10 or 11 or 12 or 13 or 14 or 15 or 16 or 17 or 18 or 19

21 Primary Health Care/

22 family nursing/ or family practice/ or general practice/ or home nursing/

23 (primary adj2 care).mp.

24 ((family adj1 practi*) or (general adj1 practi*)).mp.

25 exp Physicians, Primary Care/ or exp Physicians, Family/

26 exp General Practitioners/

27 (physician? adj (family or general)).mp.

28 21 or 22 or 23 or 24 or 25 or 26 or 27

29 community health nursing/ or community mental health services/ or Community Health Centers/ or Community medicine/ or Community psychiatry/ or Home Care Services/ or Community Health Services/

30 (community-based or community?based or "community based").ti,ab.

31 (home-based or home?based or "home based").ti,ab.

32 (community adj3 (health or service? or center? or medicine or psychiatry)).ti,ab.

33 (community-dwelling or community?dwelling or "community dwelling").ti,ab.

34 (homebound or (living adj home)).ti,ab.

35 29 or 30 or 31 or 32 or 33 or 34

36 28 or 35

37 20 or 36

38 5 and 37

39 randomized controlled trial.pt.

40 controlled clinical trial.pt.

41 placebo.ti,ab.

42 randomized.ab.

43 randomly.ab.

44 trial.ti.

45 clinical trials as topic.sh.

46 39 or 40 or 41 or 42 or 43 or 44 or 45

47 exp animals/ not humans.sh.

48 46 not 47

49 38 and 48

50 limit 49 to yr="1995 -Current"

***************************

# Appendix C: Detailed origin, transformation or imputation of reported data

A systematic approach to data collection, transformation and imputation was followed, as recommended in the Data extraction for complex meta-analysis (DECiMAL) guide ^(2)^.

Data transformation consisted of simple algebraic transformation: addition of two types of admissions representing total hospital admissions, transformation from percentage to fraction, from monthly data over a time period to total data at this time period end point, from standard error to standard deviation, from mean number of event for the subsample that had at least one event to the mean number of event for the total sample irrespective whether participants had any event.

Data imputation consisted of imputation of missing variance from the weighted average of the available variances, weighted by study sample size.

# Table A: Origin, transformation or imputation of data for proportions of persons having at least one Emergency Department visit

| **Studies** | **12 months** | **Longest follow-up** |
| --- | --- | --- |
| Challis 2004  ^(3)^ | x | Data published in the article: Challis Age Ageing 2004 and confirmed by the author |
| Chien 2008  ^(4)^ | Additional data sent by the author. Data was given in percentage, numerators were calculated with the denominators given in correspondence | Additional data sent by the author. Data was given in percentage, numerators were calculated with the denominators given in correspondence |
| Chien 2011  ^(5)^ | Additional data sent by the author. Data was given in percentage, numerators were calculated with the denominators given in correspondence | Additional data sent by the author. Data was given in percentage, numerators were calculated with the denominators given in correspondence |
| Duru 2009  ^(6,7)^ | x | Data published in the article: Duru Am J Manag Care 2009. Denominators from the article (Table 2) |
| Meeuwsen 2013  ^(8–10)^ | Additional data sent by the author. Data was given in percentage, numerators were calculated with denominators published in the article | Additional data sent by the author. Data was given in percentage, numerators were calculated with denominators published in the article |
| Nichols 2017  ^(11,12)^ | Additional data sent by the author. Denominators given in correspondence. | Additional data sent by the author. Denominators given in correspondence. |
| Samus 2014  ^(13,14)^ | x | Additional data sent by author. Denominators from the data published in the article: Amjad Health services Research 2017. |
| Sogaard 2014  ^(15–17)^ | x | Additional data sent by the author. Denominators given in correspondence |
| Thyrian 2017  ^(18–20)^ | Additional data sent by the author. Denominators given in correspondence. | Additional data sent by the author. Denominators given in correspondence. |

# Table B: Origin, transformation or imputation of data for mean number of Emergency Department visit

| **Studies** | **12 months** | **Longest follow-up** |
| --- | --- | --- |
| Bass 2003  ^(21,22)^ | Data published in article: Bass The Gerontologist 2003. Denominators calculated from the proportion of individuals randomized in each group from article. | Data published in article: Bass The Gerontologist 2003. Denominators calculated from the proportion of individuals randomized in each group from article. |
| Challis 2004  ^(3)^ | x | Data transformed from the mean number of visits in subsample and rate of visits (from article Challis Age Aging 2004 and confirmed by author), Standard Deviation (SD) imputed from the other SDs for this outcome |
| Duru 2009  ^(6,7)^ | x | Calculated from the monthly data given in article (Duru Am J Manag Care 2009) and the rate.  SD imputed from the other SDs for this outcome. Denominators from the article (Table 2) |
| Nichols 2017  ^(11,12)^ | Additional data sent by the author. Denominators given in correspondence. | Additional data sent by the author. Denominators given in correspondence. |
| Samus 2014  ^(13,14)^ | x | Data published in Amjad Health Services Research 2017. Standard error provided, SD calculated by multiplying by square root of the number of patients in the group. Denominators published in article |
| Sogaard 2014  ^(15–17)^ | x | Data published in article Sogaard Dement Geriatr Cogn Disord 2014. Standard error provided, SD calculated by multiplying by square root of the number of patients in the group. Denominators published in the article |
| Thyrian 2017  ^(18–20)^ | Additional data sent by the author. Denominators given in correspondence. | Additional data sent by the author. Denominators given in correspondence. |

# Table C: Origin, transformation or imputation of data for proportions of persons having at least one hospital admission

| **Studies** | **12 months** | **Longest follow-up** |
| --- | --- | --- |
| Callahan 2006  ^(23)^ | Data published in article Callahan JAMA 2006. Data in the article given in percentages, the numerators were calculated from the denominators, with denominators = randomized individuals, as done by the author in correspondence (for additional data for mean hospital days) | Data published in article Callahan JAMA 2006. Data in the article given in percentages, the numerators were calculated from the denominators, with denominators = randomized individuals, as done by the author in correspondence (for additional data for mean hospital days) |
| Challis 2004  ^(3)^ | x | Data published in an article : Challis Age Ageing 2004 and confirmed by the author |
| Chien 2008  ^(4)^ | Additional data sent by the author. Data was given in percentage, numerators were calculated with the denominators given in correspondence | Additional data sent by the author. Data was given in percentage, numerators were calculated with the denominators given in correspondence |
| Chien 2011  ^(5)^ | Additional data sent by the author. Data was given in percentage, numerators were calculated with the denominators given in correspondence | Additional data sent by the author. Data was given in percentage, numerators were calculated with the denominators given in correspondence |
| Duru 2009  ^(6,7)^ | x | Data published in the article: Duru Am J Manag Care 2009. Denominators from the article (Table 2) |
| Eloniemi-Sulkava 2009  ^(24)^ | x | Additional data sent by the author (Pitkala). Denominators in author correspondence. Also corresponds to the denominators found in article (Table 2 – results) |
| Joling 2013  ^(25–27)^ | Additional data sent by the author. Denominators given in correspondence. | Additional data sent by the author. Denominators given in correspondence. |
| Laakonen 2016  ^(28,29)^ | x | Additional data sent by the author. Intervention and control groups were identified according to the article data (sample of 67 in intervention group and sample of 69 in control group –denominators given in article were used) |
| Meeuwsen 2013  ^(8–10)^ | Data published in the article: Meuwsen Plos One 2013. Denominators published in the article | Data published in the article: Meuwsen Plos One 2013. Denominators published in the article |
| Menn 2012  ^(30–32)^ | Additional data sent by the author. Denominators given in correspondence. | Additional data sent by the author. Denominators given in correspondence. |
| Nichols 2017  ^(11,12)^ | Additional data sent by the author. Denominators given in correspondence. | Additional data sent by the author. Denominators given in correspondence. |
| Rubenstein 2007  ^(33)^ | Data published in an article: Rubenstein JAGS 2007. Denominators taken from the published flowchart of participants for the specific time point | Data published in an article: Rubenstein JAGS 2007. Denominators taken from the published flowchart of participants for the specific time point |
| Samus 2014  ^(13,14)^ | x | Additional data sent by author, denominators from the data published in the article: Amjad Health services Research 2017 |
| Sogaard 2014  ^(15–17)^ | x | Additional data sent by the author, denominators given in correspondence |
| Thyrian 2017  ^(18–20)^ | Additional data sent by the author. Denominators given in correspondence. | Additional data sent by the author. Denominators given in correspondence. |

# Table D: Origin, transformation or imputation of data for mean number of hospital admission

| **Studies** | **12 months** | **Longest follow-up** |
| --- | --- | --- |
| Bass 2003  ^(21,22)^ | Data published in an article : Bass The Gerontologist 2003. Denominators were calculated from the proportion of individuals randomized in each group written in article. | Data published in an article : Bass The Gerontologist 2003. Denominators were calculated from the proportion of individuals randomized in each group written in article. |
| Chien 2008  ^(4)^ | Calculated from additional data sent by the author (mean number of hospitalizations of the subgroup and rate of hospitalization). SD was imputed from the other SDs for this outcome | Calculated from additional data sent by the author (mean number of hospitalizations of the subgroup and rate of hospitalization). SD was imputed from the other SDs for this outcome |
| Chien 2011  ^(5)^ | Calculated from additional data sent by the author (mean number of hospitalizations of the subgroup and rate of hospitalization). SD was imputed from the other SDs for this outcome | Calculated from additional data sent by the author (mean number of hospitalizations of the subgroup and rate of hospitalization). SD was imputed from the other SDs for this outcome |
| Duru 2009  ^(6,7)^ | x | Calculated from the monthly data given in article (Duru Am J Manag Care 2009) and the rate.  SD was imputed from the other SDs for this outcome.  Denominators from the article (Table 2) |
| Menn 2012  ^(30–32)^ | Additional data sent by the author. Denominators given in correspondence. | Additional data sent by the author. Denominators given in correspondence. |
| Nichols 2017  ^(11,12)^ | Additional data sent by the author. Denominators given in correspondence. | Additional data sent by the author. Denominators given in correspondence. |
| Samus 2014  ^(13,14)^ | x | Data published in Amjad Health services Research 2017. Standard error provided, SD calculated by multiplying by square root of the number of patients in the group. Denominators given in article |
| Sogaard 2014  ^(15–17)^ | x | Data published in article Sogaard Dement Geriatr Cogn Disord 2014. Standard error provided, SD calculated by multiplying by square root of the number of patients in the group. Denominators published in the article |
| Thyrian 2017  ^(18–20)^ | Additional data sent by the author. Denominators given in correspondence. | Additional data sent by the author. Denominators given in correspondence. |
| Wray 2010  ^(34)^ | Calculated from the 0-6 months and 6-12 months data on both "acute admission" and "ICU admission" given in article (Wray The Gerontologist 2010). Denominators are the numbers of randomized individuals (no other available data – data from administrative source). SD was imputed from the other SDs for this outcome | Calculated from the 0-6 months and 6-12 months data on both "acute admission" and "ICU admission" given in article (Wray The Gerontologist 2010). Denominators are the numbers of randomized individuals (no other available data – data from administrative source). SD was imputed from the other SDs for this outcome |

# Table E: Origin, transformation or imputation of data for mean number of hospital days

| **Studies** | **12 months** | **Longest follow-up** |
| --- | --- | --- |
| Callahan 2006  ^(23)^ | Data published in the article Callahan JAMA 2006, SD additional data sent by the author. The denominators are the numbers of randomized individuals, as done by the author in correspondence (for additional data for mean hospital days) | Data published in the article Callahan JAMA 2006, SD additional data sent by the author. The denominators are the numbers of randomized individuals, as done by the author in correspondence (for additional data for mean hospital days) |
| Challis 2004  ^(3)^ | x | Data transformed from the mean number of visits in subsample and rate of visits (from article Challis Age Aging 2004 and confirmed by author), SD imputed. |
| Chien 2008  ^(4)^ | Calculated from additional data sent by the author (mean number of hospital days of the subgroup and rate of hospitalization). SD was imputed from the other SDs for this outcome | Calculated from additional data sent by the author (mean number of hospital days of the subgroup and rate of hospitalization). SD was imputed from the other SDs for this outcome |
| Chien 2011  ^(5)^ | Calculated from additional data sent by the author (mean number of hospital days of the subgroup and rate of hospitalization). SD was imputed from the other SDs for this outcome | Calculated from additional data sent by the author (mean number of hospital days of the subgroup and rate of hospitalization). SD was imputed from the other SDs for this outcome |
| Eloniemi-Sulkava 2009  ^(24)^ | x | Additional data sent by the author (Pitkala). Denominators in author correspondence. Also corresponds to the denominators found in article (Table 2 – results) |
| Joling 2013  ^(25–27)^ | Additional data sent by the author. Denominators given in correspondence. | Additional data sent by the author. Denominators given in correspondence. |
| Laakonen 2016  ^(28,29)^ | X | Additional data sent by the author. Intervention and control groups were identified according to the article data (sample of 67 in intervention group and sample of 69 in control group – these denominators given in article were used). Total number of days available in the article. |
| Meeuwsen 2013  ^(8–10)^ | Additional data sent by the author. Denominators published in the article | Additional data sent by the author. Denominators published in the article |
| Menn 2012  ^(30–32)^ | Additional data sent by the author. Denominators given in correspondence. | Additional data sent by the author. Denominators given in correspondence. |
| Nichols 2017  ^(11,12)^ | Additional data sent by the author. Denominators given in correspondence. | Additional data sent by the author. Denominators given in correspondence. |
| Rubenstein 2007  ^(33)^ | Data published in an article : Rubenstein JAGS 2007  Denominators taken from the published flowchart of participants for the specific time point | Data published in an article : Rubenstein JAGS 2007  Denominators taken from the published flowchart of participants for the specific time point |
| Samus 2014  ^(13,14)^ | x | Data published in Amjad Health services Research 2017. Standard error provided, SD calculated by multiplying by square root of the number of patients in the group. Denominators given in article |
| Sogaard 2014  ^(15–17)^ | x | Data published in article Sogaard Dement Geriatr Cogn Disord 2014. Standard error provided, SD calculated by multiplying by square root of the number of patients in the group. Denominators published in the article |
| Thyrian 2017  ^(18–20)^ | Additional data sent by the author. Denominators given in correspondence. | Additional data sent by the author. Denominators given in correspondence. |
| Wray 2010  ^(34)^ | Calculated from the 0-6 months and 6-12 months data given on both "acute bed days" and "ICU bed days" in article (Wray The Gerontologist 2010). Denominators are the numbers of randomized individuals (no other available data – data from administrative source). SD was imputed from the other SDs for this outcome | Calculated from the 0-6 months and 6-12 months data given on both "acute bed days" and "ICU bed days" in article (Wray The Gerontologist 2010). Denominators are the numbers of randomized individuals (no other available data – data from administrative source). SD was imputed from the other SDs for this outcome |

# Fig A. Proportion of persons having at least one Emergency Department visit (Risk Ratio and Risk Difference).

A

**Study**

**Random effects model**

**Prediction interval**

Heterogeneity:

*I*

2

= 5%

,

τ

2

= 0.0033

,

*p*

= 0.38

Chien 2008

Chien 2011

Meeuwsen 2013

Nichols 2017

Thyrian 2017

**Events**

8

8

19

55

60

**Total**

**522**

43

46

83

98

252

**Experimental**

**Events**

11

10

21

44

19

**Total**

**373**

43

46

77

99

108

**Control**

0.5

1

2

**Risk Ratio**

**RR**

**1.12**

0.73

0.80

0.84

1.26

1.35

**95%-CI**

**[0.91; 1.39]**

**[0.76; 1.67]**

[0.32; 1.63]

[0.35; 1.84]

[0.49; 1.44]

[0.95; 1.67]

[0.85; 2.15]

**Weight**

**100.0%**

7.0%

6.5%

15.4%

50.7%

20.4%

Heterogeneity:

*I*

2

= 23%

,

τ

2

= 0.0014

,

*p*

= 0.27

-0.2

-0.1

0

0.1

0.2

**Risk Difference**

**RD**

**0.02**

-0.07

-0.04

-0.04

0.12

0.06

**95%-CI**

**[-0.05; 0.09]**

**[-0.14; 0.18]**

[-0.24; 0.10]

[-0.21; 0.12]

[-0.18; 0.09]

[-0.02; 0.26]

[-0.03; 0.15]

**Weight**

**100.0%**

12.8%

14.5%

19.6%

18.6%

34.5%

B

**Study**

**Random effects model**

**Prediction interval**

Heterogeneity:

*I*

2

= 0%

,

τ

2

= 0

,

*p*

= 0.45

Challis 2004 - 6 months

Meeuwsen 2013 - 12 months

Nichols 2017 - 12 months

Thyrian 2017 - 12 months

Duru 2009 - 18 months

Samus 2014 - 18 months

Chien 2008 - 24 months

Chien 2011 - 24 months

Sogaard 2014 - 36 months

**Events**

9

19

55

60

96

41

7

7

71

**Total**

**1092**

129

83

98

252

170

110

42

45

163

**Experimental**

**Events**

8

21

44

19

66

68

14

10

73

**Total**

**984**

127

77

99

108

126

193

42

45

167

**Control**

0.5

1

2

**Risk Ratio**

**RR**

**1.06**

1.11

0.84

1.26

1.35

1.08

1.06

0.50

0.70

1.00

**95%-CI**

**[0.95; 1.19]**

**[0.92; 1.22]**

[0.44; 2.78]

[0.49; 1.44]

[0.95; 1.67]

[0.85; 2.15]

[0.87; 1.33]

[0.78; 1.44]

[0.22; 1.11]

[0.29; 1.68]

[0.78; 1.27]

**Weight**

**100.0%**

1.6%

4.7%

17.1%

6.3%

29.9%

14.2%

2.1%

1.8%

22.4%

Heterogeneity:

*I*

2

= 12%

,

τ

2

= 0.0004

,

*p*

= 0.33

-0.3

-0.2

-0.1

0

0.1

0.2

0.3

**Risk Difference**

**RD**

**0.01**

0.01

-0.04

0.12

0.06

0.04

0.02

-0.17

-0.07

-0.00

**95%-CI**

**[-0.03; 0.05]**

**[-0.06; 0.08]**

[-0.05; 0.07]

[-0.18; 0.09]

[-0.02; 0.26]

[-0.03; 0.15]

[-0.07; 0.16]

[-0.09; 0.13]

[-0.35; 0.02]

[-0.23; 0.09]

[-0.11; 0.11]

**Weight**

**100.0%**

27.9%

7.6%

7.2%

15.6%

10.1%

10.4%

4.3%

5.4%

11.4%

# Fig B. Proportion of persons having at least one hospital admission (Risk Ratio and Risk Difference).

Heterogeneity:

*I*

2

= 15%

,

τ

2

= 0.0005

,

*p*

= 0.31

-0.2

-0.1

0

0.1

0.2

**Risk Difference**

**RD**

**-0.01**

0.04

-0.09

-0.02

-0.04

0.09

-0.04

0.03

0.03

-0.07

**95%-CI**

**[-0.05; 0.03]**

**[-0.08; 0.06]**

[-0.09; 0.17]

[-0.28; 0.09]

[-0.17; 0.13]

[-0.15; 0.08]

[-0.02; 0.20]

[-0.14; 0.06]

[-0.10; 0.17]

[-0.05; 0.10]

[-0.14; 0.00]

**Weight**

**100.0%**

8.0%

4.2%

6.0%

9.6%

10.7%

12.2%

7.2%

20.5%

21.6%

**Study**

**Random effects model**

**Prediction interval**

Heterogeneity:

*I*

2

= 23%

,

τ

2

= 0.01

,

*p*

= 0.24

Callahan 2006

Chien 2008

Chien 2011

Joling 2013

Meeuwsen 2013

Menn 2012

Nichols 2017

Rubenstein 2007

Thyrian 2017

**Events**

19

9

7

12

16

97

40

210

15

**Total**

**1234**

84

43

46

78

83

216

98

334

252

**Experimental**

**Events**

13

13

8

16

8

82

37

217

14

**Total**

**1053**

69

43

46

84

77

167

99

360

108

**Control**

0.5

1

2

**Risk Ratio**

**RR**

**0.98**

1.20

0.69

0.88

0.81

1.86

0.91

1.09

1.04

0.46

**95%-CI**

**[0.85; 1.13]**

**[0.73; 1.31]**

[0.64; 2.25]

[0.33; 1.45]

[0.35; 2.21]

[0.41; 1.60]

[0.84; 4.09]

[0.74; 1.13]

[0.77; 1.55]

[0.93; 1.17]

[0.23; 0.92]

**Weight**

**100.0%**

4.8%

3.6%

2.3%

4.2%

3.2%

24.9%

13.1%

40.0%

4.0%

A

B

**Study**

**Random effects model**

**Prediction interval**

Heterogeneity:

*I*

2

= 48%

,

τ

2

= 0.0238

,

*p*

= 0.02

Challis 2004 - 6 months

Joling 2013 - 12 months

Meeuwsen 2013 - 12 months

Nichols 2017 - 12 months

Thyrian 2017 - 12 months

Callahan 2006 - 18 months

Duru 2009 - 18 months

Samus 2014 - 18 months

Chien 2008 - 24 months

Chien 2011 - 24 months

Eloniemi-Sulkava 2009 - 24 months

Laakonnen 2016 - 24 months

Menn 2012 - 24 months

Rubenstein 2007 - 36 months

Sogaard 2014 - 36 months

**Events**

25

12

16

40

15

25

53

40

8

6

31

22

144

159

87

**Total**

**1852**

129

78

83

98

252

84

170

110

42

45

63

67

216

252

163

**Experimental**

**Events**

31

16

8

37

14

17

41

58

14

8

30

21

118

131

95

**Total**

**1715**

127

84

77

99

108

69

126

193

42

45

62

69

167

280

167

**Control**

0.5

1

2

**Risk Ratio**

**RR**

**1.01**

0.79

0.81

1.86

1.09

0.46

1.21

0.96

1.21

0.57

0.75

1.02

1.08

0.94

1.35

0.94

**95%-CI**

**[0.89; 1.15]**

**[0.71; 1.45]**

[0.50; 1.27]

[0.41; 1.60]

[0.84; 4.09]

[0.77; 1.55]

[0.23; 0.92]

[0.71; 2.05]

[0.68; 1.34]

[0.87; 1.68]

[0.27; 1.22]

[0.28; 1.99]

[0.71; 1.46]

[0.66; 1.77]

[0.82; 1.08]

[1.15; 1.58]

[0.77; 1.14]

**Weight**

**100.0%**

5.3%

2.9%

2.3%

7.7%

2.9%

4.4%

8.0%

8.3%

2.5%

1.6%

7.4%

4.9%

14.9%

14.2%

12.7%

Heterogeneity:

*I*

2

= 51%

,

τ

2

= 0.0034

,

*p*

= 0.01

-0.3

-0.2

-0.1

0

0.1

0.2

0.3

**Risk Difference**

**[-0.13; 0.14]**

**0.00**

**[-0.04; 0.05]**

**100.0%**

**RD**

-0.05

-0.04

0.09

0.03

-0.07

0.05

-0.01

0.06

-0.14

-0.04

0.01

0.02

-0.04

0.16

-0.04

**95%-CI**

[-0.15; 0.05]

[-0.15; 0.08]

[-0.02; 0.20]

[-0.10; 0.17]

[-0.14; 0.00]

[-0.09; 0.19]

[-0.12; 0.09]

[-0.05; 0.17]

[-0.33; 0.04]

[-0.19; 0.11]

[-0.17; 0.18]

[-0.13; 0.18]

[-0.13; 0.05]

[ 0.08; 0.25]

[-0.14; 0.07]

**Weight**

7.7%

6.8%

7.2%

5.7%

10.0%

5.5%

7.3%

7.1%

3.8%

5.1%

4.1%

4.8%

8.3%

9.0%

7.3%

# Fig C. Mean number of Emergency Department visit (Mean Difference).

**Study**

**Random effects model**

**Prediction interval**

Heterogeneity:

*I*

2

= 31%

,

τ

2

= 0.009

,

*p*

= 0.23

Bass 2003

Nichols 2017

Thyrian 2017

**Total**

**422**

72

98

252

**Mean**

0.51

1.15

0.34

**SD**

0.980

1.460

0.045

**Experimental**

**Total**

**255**

48

99

108

**Mean**

0.68

0.88

0.19

**SD**

1.120

1.296

0.042

**Control**

-1.5

-1

-0.5

0

0.5

1

1.5

**Mean Difference**

**MD**

**0.12**

-0.17

0.27

0.14

**95%-CI**

**[-0.04; 0.28]**

**[-1.47; 1.70]**

[-0.56; 0.22]

[-0.11; 0.66]

[ 0.13; 0.15]

**Weight**

**100.0%**

13.5%

13.7%

72.8%

A

B

**Study**

**Random effects model**

**Prediction interval**

Heterogeneity:

*I*

2

= 34%

,

τ

2

= 0.0088

,

*p*

= 0.17

Challis 2004 - 6 months

Bass 2003 - 12 months

Nichols 2017 - 12 months

Thyrian 2017 - 12 months

Duru 2009 - 18 months

Samus 2014 - 18 months

Sogaard 2014 - 36 months

**Total**

**994**

129

72

98

252

170

110

163

**Mean**

0.07

0.51

1.15

0.34

0.81

0.77

0.83

**SD**

1.370

0.980

1.460

0.045

1.370

1.470

1.400

**Experimental**

**Total**

**868**

127

48

99

108

126

193

167

**Mean**

0.31

0.68

0.88

0.19

0.66

0.80

0.86

**SD**

1.560

1.120

1.296

0.042

1.560

1.670

1.680

**Control**

-0.6

-0.4

-0.2

0

0.2

0.4

0.6

**Mean Difference**

**MD**

**0.06**

-0.24

-0.17

0.27

0.14

0.15

-0.03

-0.03

**95%-CI**

**[-0.06; 0.18]**

**[-0.23; 0.35]**

[-0.60; 0.12]

[-0.56; 0.22]

[-0.11; 0.66]

[ 0.13; 0.15]

[-0.19; 0.49]

[-0.39; 0.33]

[-0.36; 0.30]

**Weight**

**100.0%**

9.3%

8.2%

8.3%

44.6%

10.1%

9.2%

10.4%

# Fig D. Mean number of hospital admission (Mean Difference).

**Study**

**Random effects model**

**Prediction interval**

Heterogeneity:

*I*

2

= 0%

,

τ

2

= 0

,

*p*

= 0.59

Bass 2003

Chien 2008

Chien 2011

Menn 2012

Nichols 2017

Thyrian 2017

Wray 2010

**Total**

**810**

72

43

46

216

98

252

83

**Mean**

0.18

0.70

0.30

0.96

0.77

0.12

0.40

**SD**

0.56

1.13

1.13

1.41

1.14

0.04

1.13

**Experimental**

**Total**

**586**

48

43

46

167

99

108

75

**Mean**

0.26

1.20

0.50

0.99

0.74

0.16

0.30

**SD**

0.59

1.20

1.20

1.33

1.22

0.44

1.20

**Control**

-0.5

0

0.5

**Mean Difference**

**MD**

**-0.05**

-0.08

-0.50

-0.20

-0.03

0.03

-0.04

0.10

**95%-CI**

**[-0.12; 0.02]**

**[-0.14; 0.04]**

[-0.29; 0.13]

[-0.99; -0.01]

[-0.68; 0.28]

[-0.31; 0.25]

[-0.30; 0.36]

[-0.12; 0.04]

[-0.26; 0.46]

**Weight**

**100.0%**

10.8%

2.0%

2.1%

6.3%

4.4%

70.8%

3.6%

A

B

**Study**

**Random effects model**

**Prediction interval**

Heterogeneity:

*I*

2

= 44%

,

τ

2

= 0.014

,

*p*

= 0.07

Bass 2003 - 12 months

Nichols 2017 - 12 months

Thyrian 2017 - 12 months

Wray 2010 - 12 months

Duru 2009 - 18 months

Samus 2014 - 18 months

Chien 2008 - 24 months

Chien 2011 - 24 months

Menn 2012 - 24 months

Sogaard 2014 - 36 months

**Total**

**1251**

72

98

252

83

170

110

42

45

216

163

**Mean**

0.18

0.77

0.12

0.40

0.56

0.56

0.50

0.20

1.70

1.42

**SD**

0.56

1.14

0.04

1.32

1.32

1.05

1.32

1.32

2.02

2.04

**Experimental**

**Total**

**1070**

48

99

108

75

126

193

42

45

167

167

**Mean**

0.26

0.74

0.16

0.30

0.88

0.57

1.40

0.60

2.02

1.46

**SD**

0.59

1.22

0.44

1.28

1.28

1.11

1.28

1.28

2.05

2.46

**Control**

-1

-0.5

0

0.5

1

**Mean Difference**

**MD**

**-0.13**

-0.08

0.03

-0.04

0.10

-0.32

-0.01

-0.90

-0.40

-0.32

-0.04

**95%-CI**

**[-0.25; 0.00]**

**[-0.43; 0.18]**

[-0.29; 0.13]

[-0.30; 0.36]

[-0.12; 0.04]

[-0.31; 0.51]

[-0.62; -0.02]

[-0.26; 0.24]

[-1.46; -0.34]

[-0.94; 0.14]

[-0.73; 0.09]

[-0.53; 0.45]

**Weight**

**100.0%**

15.3%

9.3%

24.8%

6.9%

10.5%

12.9%

4.1%

4.4%

6.7%

5.2%

# Fig E. Mean number of hospital days (Mean Difference).

**Study**

**Random effects model**

**Prediction interval**

Heterogeneity:

*I*

2

= 92%

,

τ

2

= 1.188

,

*p*

< 0.01

Callahan 2006

Chien 2008

Chien 2011

Joling 2013

Meeuwsen 2013

Menn 2012

Nichols 2017

Rubenstein 2007

Thyrian 2017

Wray 2010

**Total**

**1317**

84

43

46

78

83

216

98

334

252

83

**Mean**

1.70

2.10

0.90

1.74

4.30

10.74

5.68

0.57

4.03

3.00

**SD**

5.20

13.18

13.18

8.14

3.00

18.18

9.85

1.20

0.86

13.18

**Experimental**

**Total**

**1128**

69

43

46

84

77

167

99

360

108

75

**Mean**

1.00

4.20

2.40

2.21

0.50

12.11

6.93

0.51

4.40

3.90

**SD**

3.40

9.17

9.17

7.25

1.50

18.63

13.13

0.93

1.43

9.17

**Control**

-6

-4

-2

0

2

4

6

**Mean Difference**

**MD**

**0.35**

0.70

-2.10

-1.50

-0.47

3.80

-1.37

-1.25

0.06

-0.37

-0.90

**95%-CI**

**[-0.59; 1.29]**

**[-2.39; 3.10]**

[-0.67; 2.07]

[-6.90; 2.70]

[-6.14; 3.14]

[-2.85; 1.91]

[ 3.07; 4.53]

[-5.09; 2.35]

[-4.49; 1.99]

[-0.10; 0.22]

[-0.66; -0.08]

[-4.41; 2.61]

**Weight**

**100.0%**

13.7%

3.2%

3.4%

8.6%

17.3%

4.8%

5.8%

19.2%

18.9%

5.2%

A

**Study**

**Random effects model**

**Prediction interval**

Heterogeneity:

*I*

2

= 88%

,

τ

2

= 1.242

,

*p*

< 0.01

Challis 2004 - 6 months

Joling 2013 - 12 months

Meeuwsen 2013 - 12 months

Nichols 2017 - 12 months

Thyrian 2017 - 12 months

Wray 2010 - 12 months

Callahan 2006 - 18 months

Samus 2014 - 18 months

Chien 2008 - 24 months

Chien 2011 - 24 months

Eloniemi-Sulkava 2009 - 24 months

Laakonnen 2016 - 24 months

Menn 2012 - 24 months

Rubenstein 2007 - 36 months

Sogaard 2014 - 36 months

**Total**

**1765**

129

78

83

98

252

83

84

110

42

45

63

67

216

252

163

**Mean**

6.01

1.74

4.30

5.68

4.03

3.00

2.60

2.96

1.80

0.80

23.30

8.20

19.88

0.55

8.48

**SD**

19.14

8.14

3.00

9.85

0.86

19.14

7.90

7.24

19.14

19.14

42.30

36.70

27.34

1.20

18.13

**Experimental**

**Total**

**1664**

127

84

77

99

108

75

69

193

42

45

62

69

167

280

167

**Mean**

6.10

2.21

0.50

6.93

4.40

3.90

1.50

2.80

5.50

2.30

28.10

12.90

23.39

0.49

7.33

**SD**

19.3

7.2

1.5

13.1

1.4

19.3

4.1

7.9

19.3

19.3

51.2

35.9

29.4

1.0

15.4

**Control**

-20

-10

0

10

20

**Mean Difference**

**MD**

**0.42**

-0.09

-0.47

3.80

-1.25

-0.37

-0.90

1.10

0.16

-3.70

-1.50

-4.80

-4.70

-3.51

0.06

1.15

**95%-CI**

**[ -0.50; 1.34]**

**[ -2.19; 3.03]**

[ -4.80; 4.62]

[ -2.85; 1.91]

[ 3.07; 4.53]

[ -4.49; 1.99]

[ -0.66; -0.08]

[ -6.90; 5.10]

[ -0.85; 3.05]

[ -1.59; 1.91]

[-11.91; 4.51]

[ -9.44; 6.44]

[-21.28; 11.68]

[-16.91; 7.51]

[ -9.27; 2.25]

[ -0.13; 0.25]

[ -2.48; 4.78]

**Weight**

**100.0%**

3.1%

8.0%

15.8%

5.5%

17.3%

2.1%

9.8%

10.7%

1.2%

1.2%

0.3%

0.5%

2.2%

17.5%

4.7%

B

# Fig F. Quality Appraisal using the Cochrane Risk of Bias Tool ^(35)^


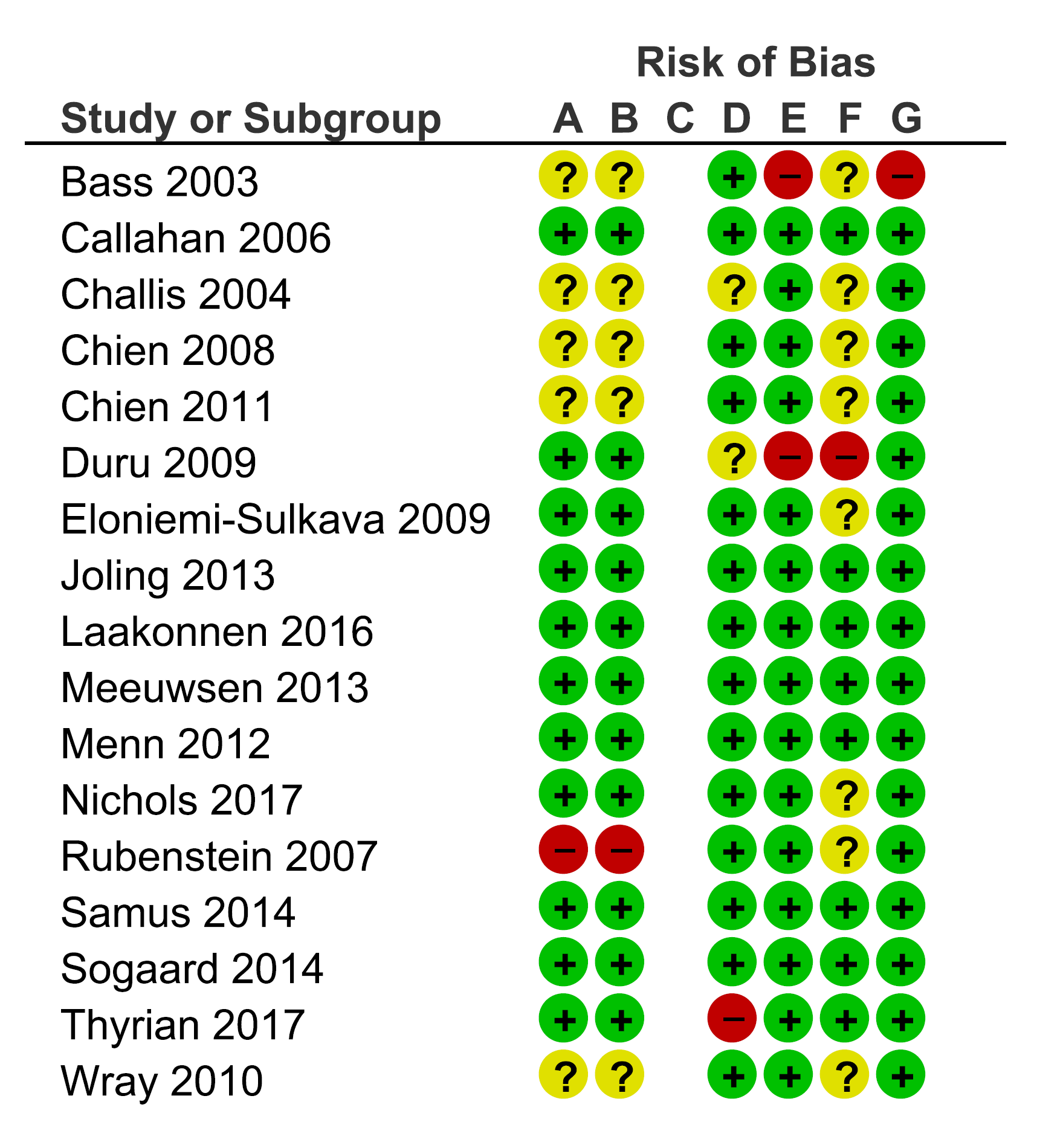


^h^

^a b c d e f g^

# References

1. Effective Practice and Organisation of Care (EPOC). EPOC Taxonomy; 2015. <https://epoc.cochrane.org/epoc-taxonomy>. Accessed September 5, 2017.

2. Pedder H, Sarri G, Keeney E, Nunes V, Dias S. Data extraction for complex meta-analysis (DECiMAL) guide. *Syst Rev*. 2016;5(1):212.

3. Challis D, Clarkson P, Williamson J, et al. The value of specialist clinical assessment of older people prior to entry to care homes. *Age Ageing*. 2004;33(1):25–34.

4. Chien WT, Lee YM. A disease management program for families of persons in Hong Kong with dementia. *Psychiatr Serv*. 2008;59(4):433–6.

5. Chien WT, Lee IYM. Randomized controlled trial of a dementia care programme for families of home-resided older people with dementia. *J Adv Nurs*. 2011;67(4):774–87.

6. Duru OK, Ettner SL, Vassar SD, Chodosh J, Vickrey BG. Cost evaluation of a coordinated care management intervention for dementia. *Am J Manag Care*. 2009;15(8):521–8.

7. Vickrey BG, Mittman BS, Connor KI, et al. The effect of a disease management intervention on quality and outcomes of dementia care: a randomized, controlled trial. *Ann Intern Med*. 2006;145(10):713–26.

8. Meeuwsen E, Melis R, van der Aa G, et al. Cost-effectiveness of one year dementia follow-up care by memory clinics or general practitioners: economic evaluation of a randomised controlled trial. *PloS One*. 2013;8(11):e79797.

9. Meeuwsen EJ, German P, Melis RJF, et al. Cost-effectiveness of post-diagnosis treatment in dementia coordinated by Multidisciplinary Memory Clinics in comparison to treatment coordinated by general practitioners: an example of a pragmatic trial. *J Nutr Health Aging*. 2009;13(3):242–8.

10. Meeuwsen EJ, Melis RJF, Van Der Aa GCHM, et al. Effectiveness of dementia follow-up care by memory clinics or general practitioners: randomised controlled trial. *BMJ*. 2012;344:e3086.

11. Nichols LO, Martindale-Adams J, Zhu CW, Kaplan EK, Zuber JK, Waters TM. Impact of the REACH II and REACH VA Dementia Caregiver Interventions on Healthcare Costs*. J Am Geriatr Soc*. 2017;65(5):931–6.

12. Belle SH, Burgio L, Burns R, et al. Enhancing the quality of life of dementia caregivers from different ethnic or racial groups: a randomized, controlled trial. *Ann Intern Med*. 2006;145(10):727–38.

13. Samus QM, Johnston D, Black BS, et al. A multidimensional home-based care coordination intervention for elders with memory disorders: the Maximizing Independence at Home (MIND) pilot randomized trial. *Am J Geriatr Psychiatry*. 2014;22(4):398–414.

14. Amjad H, Wong SK, Roth DL, et al. Health Services Utilization in Older Adults with Dementia Receiving Care Coordination: The MIND at Home Trial. [published online January 12, 2017]. *Health Serv Res*. doi: 10.1111/1475-6773.12647.

15. Søgaard R, Sørensen J, Waldorff FB, Eckermann A, Buss DV, Waldemar G. Cost analysis of early psychosocial intervention in Alzheimer’s disease. *Dement Geriatr Cogn Disord*. 2014;37(3–4):141–53.

16. Søgaard R, Sørensen J, Waldorff FB, et al. Early psychosocial intervention in Alzheimer’s disease: cost utility evaluation alongside the Danish Alzheimer’s Intervention Study (DAISY). *BMJ Open*. 2014;4(1):e004105.

17. Waldemar G, Waldorff FB, Buss DV, et al. The Danish Alzheimer intervention study: rationale, study design and baseline characteristics of the cohort. *Neuroepidemiology*. 2011;36(1):52–61.

18. Thyrian JR, Hertel J, Wucherer D, et al. Effectiveness and Safety of Dementia Care Management in Primary Care: A Randomized Clinical Trial. *JAMA Psychiatry*. 2017;74(10):996-1004. doi: 10.1001/jamapsychiatry.2017.2124.

19. Thyrian JR, Fiß T, Dreier A, et al. Life- and person-centred help in Mecklenburg-Western Pomerania, Germany (DelpHi): study protocol for a randomised controlled trial. *Trials*. 2012;13:56.

20. Dreier A, Thyrian JR, Eichler T, Hoffmann W. Qualifications for nurses for the care of patients with dementia and support to their caregivers: A pilot evaluation of the dementia care management curriculum. *Nurse Educ Today*. 2016;36:310–7.

21. Clark PA, Bass DM, Looman WJ, McCarthy CA, Eckert S. Outcomes for patients with dementia from the Cleveland Alzheimer’s Managed Care Demonstration. *Aging Ment Health*. 2004;8(1):40–51.

22. Bass DM, Clark PA, Looman WJ, McCarthy CA, Eckert S. The Cleveland Alzheimer’s managed care demonstration: outcomes after 12 months of implementation. *Gerontologist.* 2003;43(1):73–85.

23. Callahan CM, Boustani MA, Unverzagt FW, et al. Effectiveness of collaborative care for older adults with Alzheimer disease in primary care: a randomized controlled trial. *JAMA*. 2006;295(18):2148–57.

24. Eloniemi-Sulkava U, Saarenheimo M, Laakkonen M-L, et al. Family care as collaboration: effectiveness of a multicomponent support program for elderly couples with dementia. Randomized controlled intervention study. *J Am Geriatr Soc*. 2009;57(12):2200–8.

25. Joling KJ, Bosmans JE, van Marwijk HWJ, et al. The cost-effectiveness of a family meetings intervention to prevent depression and anxiety in family caregivers of patients with dementia: a randomized trial. *Trials*. 2013;14:305.

26. Joling KJ, van Hout HPJ, Scheltens P, et al. (Cost)-effectiveness of family meetings on indicated prevention of anxiety and depressive symptoms and disorders of primary family caregivers of patients with dementia: design of a randomized controlled trial. *BMC Geriatr*. 2008;8:2.

27. Joling KJ, van Marwijk HWJ, Smit F, et al. Does a family meetings intervention prevent depression and anxiety in family caregivers of dementia patients? A randomized trial. *PloS One.* 2012;7(1):e30936.

28. Laakkonen M-L, Kautiainen H, Hölttä E, et al. Effects of Self-Management Groups for People with Dementia and Their Spouses-Randomized Controlled Trial. *J Am Geriatr Soc*. 2016;64(4):752–60.

29. Laakkonen M-L, Savikko N, Hölttä E, et al. Self-management groups for people with dementia and their spousal caregivers. A randomized, controlled trial. Baseline findings and feasibility. *Eur Geriatr Med.* 2013;4(6):389–93.

30. Menn P, Holle R, Kunz S, et al. Dementia care in the general practice setting: a cluster randomized trial on the effectiveness and cost impact of three management strategies. *Value Health.* 2012;15(6):851–9.

31. Schwarzkopf L, Menn P, Kunz S, et al. Costs of care for dementia patients in community setting: an analysis for mild and moderate disease stage. *Value Health.* 2011;14(6):827–35.

32. Holle R, Grässel E, Ruckdäschel S, et al. Dementia care initiative in primary practice: study protocol of a cluster randomized trial on dementia management in a general practice setting. *BMC Health Serv Res.* 2009;9:91.

33. Rubenstein LZ, Alessi CA, Josephson KR, Trinidad Hoyl M, Harker JO, Pietruszka FM. A randomized trial of a screening, case finding, and referral system for older veterans in primary care. *J Am Geriatr Soc.* 2007;55(2):166–74.

34. Wray LO, Shulan MD, Toseland RW, Freeman KE, Vásquez BE, Gao J. The effect of telephone support groups on costs of care for veterans with dementia. *Gerontologist.* 2010;50(5):623–31.

35. Higgins JPT, Altman DG, Gøtzsche PC, et al. The Cochrane Collaboration’s tool for assessing risk of bias in randomised trials. *BMJ.* 2011;343:d5928.
