# Supplementary material for: Impact of health service interventions on acute hospital use in community-dwelling persons with dementia: A systematic literature review and meta-analysis
Source: PLoS One. 2019 Jun 21;14(6):e0218426. doi: 10.1371/journal.pone.0218426 (PMC6588225; doi:10.1371/journal.pone.0218426)
Supplement: S2 File — (DOCX) [file pone.0218426.s002.docx]

# S2 File. PRISMA Checklist

| **Section/topic** | **#** | **Checklist item** | **Reported on page #** |
| --- | --- | --- | --- |
| **TITLE** | | |  |
| Title | 1 | Identify the report as a systematic review, meta-analysis, or both. | #1 |
| “Impact of health service interventions on acute hospital use in community-dwelling persons with dementia A systematic literature review and meta-analysis” | | | |
| **ABSTRACT** | | |  |
| Structured summary | 2 | Provide a structured summary including, as applicable: background; objectives; data sources; study eligibility criteria, participants, and interventions; study appraisal and synthesis methods; results; limitations; conclusions and implications of key findings; systematic review registration number. | #2, 3  See Abstract. |
| **INTRODUCTION** | | |  |
| Rationale | 3 | Describe the rationale for the review in the context of what is already known. | #4 |
| “Persons with dementia have twice the acute hospital use as older persons without dementia. (…) Reducing avoidable acute hospital use in persons with dementia is thus a global healthcare priority. (…) To date, there is no comprehensive evidence synthesis or meta-analysis on the impact of health service interventions on acute hospital use in community-dwelling persons with dementia” | | | |
| Objectives | 4 | Provide an explicit statement of questions being addressed with reference to participants, interventions, comparisons, outcomes, and study design (PICOS). | #2, 5 |
| “The aim of this systematic review and meta-analysis was to synthesize available evidence on the impact of health service interventions on acute hospital use in community-dwelling persons with dementia compared to usual care.“ | | | |
| **METHODS** | | |  |
| Protocol and registration | 5 | Indicate if a review protocol exists, if and where it can be accessed (e.g., Web address), and, if available, provide registration information including registration number. | #3, 5 |
| “We followed an a priori registered protocol (PROSPERO ID: CRD42016046444)” | | | |
| Eligibility criteria | 6 | Specify study characteristics (e.g., PICOS, length of follow-up) and report characteristics (e.g., years considered, language, publication status) used as criteria for eligibility, giving rationale. | #5  Appendix A in S1 File: Detailed eligibility criteria of intervention |
| “**Eligibility criteria** Published articles on RCTs measuring the impact of health service interventions on acute hospital use in community-dwelling persons with dementia were included. **Population:** Community-dwelling persons with dementia or their caregivers or both. **Intervention:** Any health service intervention as defined by the Effective Practice and Organization of Care Cochrane Group (EPOC) Taxonomy 2015: “delivery arrangements”, “financial arrangements”, “governance arrangements”, or “implementation strategies” (Detailed eligibility criteria of interventions in Appendix A in S1 File) [24]. **Comparison:** Usual care. **Outcomes:** Proportion or mean number of ED visits, proportion or mean number of hospital admissions, mean number of hospital days, in persons with dementia. Eligibility was restricted to interventions in high-income countries [25].”  “We searched for publications in English or French in four databases (…) from January 1995 (first publications on health service interventions for persons living with chronic diseases ^(5)^) to August 2017.” | | | |
| Information sources | 7 | Describe all information sources (e.g., databases with dates of coverage, contact with study authors to identify additional studies) in the search and date last searched. | #5, 6 |
| “We searched for publications (…) in four databases: MEDLINE (“In-Process & Other Non-Indexed Citations”), EMBASE, CINAHL, and Cochrane Central Register of Controlled Trials (CENTRAL) from January 1995 (first publications on health service interventions for persons living with chronic diseases ^(5)^) to August 2017.”  “The search was expanded using backward citation tracking in the reference list of included articles and recent systematic reviews on the topic [14–20] and forward citation tracking of all included studies using Scopus. Authors of relevant trials measuring impact on healthcare costs were inquired about data on acute hospital uses.” | | | |
| Search | 8 | Present full electronic search strategy for at least one database, including any limits used, such that it could be repeated. | Appendix B in S1 File: Medline full electronic search strategy |
| Study selection | 9 | State the process for selecting studies (i.e., screening, eligibility, included in systematic review, and, if applicable, included in the meta-analysis). | #6 |
| “Reviewers (CGS, MT, ML) independently assessed all records for eligibility. Disagreements were resolved by consensus or a third reviewer (IV).” | | | |
| Data collection process | 10 | Describe method of data extraction from reports (e.g., piloted forms, independently, in duplicate) and any processes for obtaining and confirming data from investigators. | #6 |
| “Two authors (CGS and ML) independently collected data on structured forms. Companion articles were used if needed, to access data on intervention details. To avoid bias due to selective inclusion of trials effect estimates, corresponding authors of included studies were contacted by email about unpublished data on outcomes at any other time point [27]. In cases of non-response, reminder emails and social media messages (ResearchGate, LinkedIn) were sent to corresponding and last authors.” | | | |
| Data items | 11 | List and define all variables for which data were sought (e.g., PICOS, funding sources) and any assumptions and simplifications made. | #6,7  Appendix C in S1 File: Detailed origin, transformation or imputation of reported data  Tables A-E in S1 File |
| “We pooled estimates for the following five outcomes at two endpoints. We pooled the estimated proportions of persons having at least one ED visit and/or at least one hospital admission, the mean number of ED visits, the mean number of hospital admissions, and the mean number of hospital days on the total sample of participants irrespective of whether participants used the corresponding service. “  “A systematic approach to data collection, transformation and imputation was followed, as recommended in the Data extraction for complex meta-analysis (DECiMAL) guide (Detailed origin, transformation or imputation of reported data in Appendix C and Tables A-E in S1 File) [26].”  “Transformation of data consisted of simple algebraic transformation. Data imputation consisted of weighted mean imputation of missing variance estimates. When the sample size in each group was not clearly stated, the randomised number of individuals in the text or flow chart determined the intention-to-treat population. Clustered randomised trials were identified and data adjusted on the clustering effect was collected. If unavailable, unadjusted data was collected.” | | | |
| Risk of bias in individual studies | 12 | Describe methods used for assessing risk of bias of individual studies (including specification of whether this was done at the study or outcome level), and how this information is to be used in any data synthesis. | #6,7 |
| “Risk of bias was rated at the study level by two independent reviewers (CGS and ML), using the Cochrane Collaboration’s tool [28]. Companion articles, especially published protocols, were used to appraise quality. Blinding of participants and personnel was not assessed due to the nature of the interventions. For the six remaining individual domains, studies were classified into low, unclear or high risk of bias according to specific criteria of the tool [28]. As recommended by the Cochrane Handbook for Systematic Reviews of Interventions, studies of low quality were not excluded, but sensitivity analyses were conducted [21]. “ | | | |
| Summary measures | 13 | State the principal summary measures (e.g., risk ratio, difference in means). | #7 |
| “Risk differences, risk ratio and mean differences were calculated to determine the average relative and absolute effect of the interventions on the dichotomous and continuous outcomes.” | | | |
| Synthesis of results | 14 | Describe the methods of handling data and combining results of studies, if done, including measures of consistency (e.g., I^2^) for each meta-analysis. | #7,8 |
| “The statistical software R, and the meta and ggplot2 packages, were used to perform analyses [29]. The unit of analysis was the unique RCT. Random-effects models were employed to allow for varying effect sizes across studies due to heterogeneity of interventions and/or study populations. Risk differences, risk ratio and mean differences were calculated to determine the average relative and absolute effect of the interventions on the dichotomous and continuous outcomes. Not every cluster RCT study had published data adjusting for a potential clustering effect. Mixing unadjusted data from cluster RCTs with data from individual RCTs can lead to artificially narrow confidence intervals [30]. The Cochrane Collaboration recommends performing an “effective sample size” calculation to pool unadjusted data from cluster RCTs and individual RCTs together. As data necessary to perform this ‘effective sample size’ calculation was not available for every cluster RCT, we performed sensitivity analyses excluding unadjusted data from cluster RCTs [30]. Heterogeneity across studies was assessed by calculating the I^2^ statistic as well as predictions intervals. Following Cochrane recommendations, our interpretation of the I^2^ statistic was that over 40% may represent moderate to considerable heterogeneity [21]. Prediction intervals are another measure of heterogeneity and are easier to interpret and relate to the clinical implication of the observed heterogeneity [31]. Prediction intervals estimate a pre-specified distribution range (here: 95%) of treatment effects that can be expected in future settings. Meta-analysis results were labelled inconclusive if the range of treatment effects consistent with the prediction interval included both positive and negative clinically relevant effects. “ | | | |
| Risk of bias across studies | 15 | Specify any assessment of risk of bias that may affect the cumulative evidence (e.g., publication bias, selective reporting within studies). | #8 |
| “We did not generate funnel plots to identify reporting bias, because interpretation would have been questionable since most of the data in the analysis was unpublished. We conducted additional sensitivity analyses investigating changes in estimated pooled effects due to removing unpublished data provided by authors from the analyses [32].” | | | |
| Additional analyses | 16 | Describe methods of additional analyses (e.g., sensitivity or subgroup analyses, meta-regression), if done, indicating which were pre-specified. | #8 |
| “Post hoc subgroup analyses were performed to explore heterogeneity for outcomes pooling a minimum of four studies and showing moderate to considerable heterogeneity (I^2^ > 40%). Criteria used to perform these subgroup analyses were the types of interventions according to the EPOC taxonomy (either the main component or one of several), country (United States vs. other), and follow-up time. The number of studies included did not allow meaningful application of meta-regression methods.  We conducted several sensitivity analyses to determine the robustness of meta-analysis results. We investigated changes in estimated pooled effects when removing: RCTs with at least one item at high risk of bias, outlying RCTs based on a graphical assessment of the corresponding forest plot, and cluster RCTs that did not properly take clustering into account.” | | | |
| **RESULTS** | | |  |
| Study selection | 17 | Give numbers of studies screened, assessed for eligibility, and included in the review, with reasons for exclusions at each stage, ideally with a flow diagram. | #8,9, Fig 1 |
| Flow diagram: “14632 records screened, 47 full texts assessed for eligibility, 17 unique RCTs included for meta-analysis” | | | |
| Study characteristics | 18 | For each study, present characteristics for which data were extracted (e.g., study size, PICOS, follow-up period) and provide the citations. | #10-14, Table 1, Table 2 |
| Table 1: Studies characteristics  “Seventeen unique trials provided data on 4,549 community-dwelling persons living with dementia (study populations ranging from 88 to 792 persons, median randomisation arm size: 96) (Table 1) (13.36–65). These persons had a mean age of 77 years (standard deviation: 4) and 49% were females (proportion ranged from 4% to 74%). Twelve of the seventeen trials reported dementia severity at baseline, ranging from mild to severe: 6/12 mild, 5/12 moderate, 1/12 moderate to severe. None of the trials reported selection of participants based on risk of acute hospital use.  Health service interventions consisted of one or a combination of the following EPOC taxonomy components: case management (9/17), self-management (13/17), comprehensive geriatric assessment (2/17), educational materials/educational meetings (healthcare professional education) (2/17), use of information and communication technology (4/17), teams (6/17), shared care (1/17), site of service delivery (memory clinic) (1/17).  Interventions were implemented and evaluated in studies conducted in the USA (7), in Europe (8) and in Hong Kong (2). Duration of interventions ranged from two to 36 months, median duration 12 months. None of the interventions involved financial or governance arrangements.“ | | | |
| Risk of bias within studies | 19 | Present data on risk of bias of each study and, if available, any outcome level assessment (see item 12). | #15, Fig F in S1 File |
| **Quality and robustness of the evidence**  Four of the 17 trials were judged as having at least one area of high risk of bias (Fig F in S1 File). Eleven trials reported adequate sequence generation, 11 trials properly concealed allocation, 14 trials implemented blinding of outcome assessors, and 15 trials adequately addressed incomplete outcome data. Only eight trials published a study protocol and reported (or provided upon request) pre-specified outcomes.” | | | |
| Results of individual studies | 20 | For all outcomes considered (benefits or harms), present, for each study: (a) simple summary data for each intervention group (b) effect estimates and confidence intervals, ideally with a forest plot. | Figs A-E in S1 File |
| Forest plots in Figs A-E in S1 File | | | |
| Synthesis of results | 21 | Present results of each meta-analysis done, including confidence intervals and measures of consistency. | Figs 2A, 2B and 3, and Figs A-E in S1 File |
| “None of the considered outcome comparisons provided conclusive evidence supporting the hypothesis that health service interventions lead to a decrease in service use as measured by ED visits, hospital admission or hospital days (Fig 2 and 3, and Figs A-E in S1 Fileappe). Furthermore, in every meta-analysis, the estimated 95% prediction intervals indicated that an important increase in service use may be associated with the interventions.” | | | |
| Risk of bias across studies | 22 | Present results of any assessment of risk of bias across studies (see Item 15). | #15 |
| “Sensitivity analyses of the impact of unpublished data provided by authors on meta-analysis results did not suggest reporting bias. “ | | | |
| Additional analysis | 23 | Give results of additional analyses, if done (e.g., sensitivity or subgroup analyses, meta-regression [see Item 16]). | #15 |
| “Post hoc subgroup analyses did not suggest any systematic dependencies of effects in relation to type of intervention, country or follow-up time.”  “Sensitivity analyses to determine the robustness of meta-analysis results did not lead to any change in estimated pooled effects that would alter the conclusions.” | | | |
| **DISCUSSION** | | |  |
| Summary of evidence | 24 | Summarize the main findings including the strength of evidence for each main outcome; consider their relevance to key groups (e.g., healthcare providers, users, and policy makers). | #15-18 |
| “The results of this systematic review and meta-analysis did not establish superiority of health service interventions over usual care to reduce acute hospital use in community-dwelling persons with dementia. There was no detectable signal favouring any type of health service intervention. Overall evidence had low risk of bias. Sensitivity analyses confirmed the robustness of the results. Furthermore, for every outcome, the estimated 95% prediction intervals indicated that an important increase in service use may be associated with these interventions.  These results are of particular interest to policy makers, persons living with dementia, their caregivers, and healthcare professionals. There is no cure or disease-modifying treatment for dementia and no promising advances in the near future ^65^. Improving healthcare delivery remains essential to limit rising acute hospital use and associated costs, improve quality of life of patients and their caregivers, and prevent adverse outcomes for persons living with dementia. The absence of evidence for an impact on acute hospital use of any type of health service intervention is thus highly concerning. The possibility that these interventions may increase acute hospital use is not to be disregarded. “ | | | |
| Limitations | 25 | Discuss limitations at study and outcome level (e.g., risk of bias), and at review-level (e.g., incomplete retrieval of identified research, reporting bias). | #18, 19 |
| “Overall evidence had low risk of bias. Sensitivity analyses confirmed the robustness of the results. “  “This is the first systematic review and meta-analysis (…) to include predominantly unpublished data provided by the authors of included randomised controlled trials. (…) These strategies dramatically increased the range of synthesised evidence and were likely to have decreased potential publication bias impact on our effect estimates [65,66]. | | | |
| Conclusions | 26 | Provide a general interpretation of the results in the context of other evidence, and implications for future research. | #19 |
| **“**With the data available, it was not possible to establish superiority of any health service intervention beyond usual care to reduce acute hospital use in community-dwelling persons with dementia. In fact, our evidence synthesis findings do not rule out the possibility that the studied health service interventions may be associated with an important increase in service use.  We have no recommendations for health service interventions to be implemented. However, we can propose a research agenda focused on: 1) development of accurate measures of potentially avoidable acute hospital use by community-dwelling persons with dementia; 2) identifying the causes and determinants of potentially avoidable acute hospital use; 3) development of a validated screening tool to target high-risk population; 4) co-design of health service interventions with patients and caregivers that address the causes of avoidable acute hospital use; and 5) rigorous testing of the impact of these co-designed interventions in high-risk community-dwelling persons with dementia.” | | | |
| **FUNDING** | | |  |
| Funding | 27 | Describe sources of funding for the systematic review and other support (e.g., supply of data); role of funders for the systematic review. | Only included in online submission system |

From: Moher D, Liberati A, Tetzlaff J, Altman DG, The PRISMA Group (2009). Preferred Reporting Items for Systematic Reviews and Meta-Analyses: The PRISMA Statement. PLoS Med 6(6): e1000097. doi:10.1371/journal.pmed1000097
